# Substrate Profiling of the Cobalt Nitrile Hydratase from *Rhodococcus rhodochrous* ATCC BAA 870

**DOI:** 10.3390/molecules25010238

**Published:** 2020-01-06

**Authors:** Adelaide R. Mashweu, Varsha P. Chhiba-Govindjee, Moira L. Bode, Dean Brady

**Affiliations:** 1 Molecular Sciences Institute, School of Chemistry, University of the Witwatersrand, Johannesburg 2050, South Africa; 1715870@students.wits.ac.za (A.R.M.); VChhiba@csir.co.za (V.P.C.-G.); 2 CSIR Chemical Production Cluster, PO Box 395, Pretoria 0001, South Africa

**Keywords:** green chemistry, nitrile hydratase, biocatalysis, carboxamide

## Abstract

The aromatic substrate profile of the cobalt nitrile hydratase from *Rhodococcus rhodochrous* ATCC BAA 870 was evaluated against a wide range of nitrile containing compounds (>60). To determine the substrate limits of this enzyme, compounds ranging in size from small (90 Da) to large (325 Da) were evaluated. Larger compounds included those with a bi-aryl axis, prepared by the Suzuki coupling reaction, Morita–Baylis–Hillman adducts, heteroatom-linked diarylpyridines prepared by Buchwald–Hartwig cross-coupling reactions and imidazo[1,2-*a*]pyridines prepared by the Groebke–Blackburn–Bienaymé multicomponent reaction. The enzyme active site was moderately accommodating, accepting almost all of the small aromatic nitriles, the diarylpyridines and most of the bi-aryl compounds and Morita–Baylis–Hillman products but not the Groebke–Blackburn–Bienaymé products. Nitrile conversion was influenced by steric hindrance around the cyano group, the presence of electron donating groups (e.g., methoxy) on the aromatic ring, and the overall size of the compound.

## 1. Introduction

Nitrile hydratase is a well-known success story for biocatalysis. The base or acid conversion of a nitrile to an amide is problematic as the subsequent conversion to the carboxylic acid is faster and thus hard to prevent. This was initially circumvented through the use of metallic copper heterogeneous catalysts and encouraged the exploration of other metal catalysts [1]. However, biocatalysts have now come to be the preferred catalysts. The nitrile hydratase-expressing organism *Rhodococcus rhodochrous* J1 was selected to convert acrylonitrile into acrylamide [2], which is now manufactured at a multi-hundred thousand ton per annum scale with near perfect chemoselectivity (>99.99%). The same organism was also found to be active against cyanopyridines and hence is used in the manufacture of nicotinamide (vitamin B3) [3]. Whole cell nitrile hydratase catalysts have also been applied in the production of 5-cyanovaleramide [4,5,6,7,8], as well as a wide range of commercial pharmaceuticals, agrochemicals and food additives [9].

**Scheme 1:**
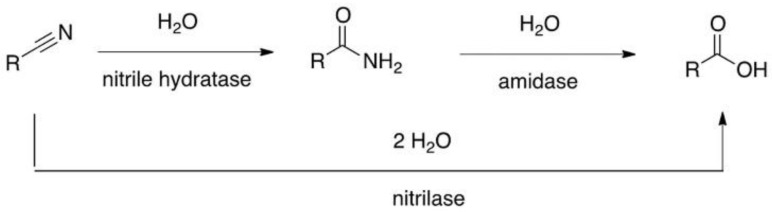
Reactions catalysed by nitrile degrading enzymes.

Nitrile hydratase (EC 4.2.1.84) is a metalloenzyme that catalyses hydration (partial hydrolysis) of nitriles to the corresponding carboxamide (Scheme 1). There are two types of nitrile hydratase enzyme—those with a non-haem iron and those with a non-corrin cobalt at the active site. Both types are found in the genus Rhodococcus, the Fe-Nhase being expressed by *R. erythropolis* and Co-Nhase being expressed by *R. rhodochrous* [10]. The cobalt NHase activity from *R. rhodochrous* J1, like that of the iron containing NHase from *R. erythropolis*, is able to hydrate aliphatic nitrile compounds but has been reported to have higher activity against aromatic compounds [11]. Apart from the nitrile hydratase and an associated amidase [12,13], Rhodococci also express a competing nitrilase that generates the corresponding carboxylic acid [14]. This creates a complication when analysing the nitrile hydratase activity using whole cells [15,16] as both pathways can result in either carboxamides or carboxylic acid products, depending on the substrate [17]. Whole cells of Rhodococci have been shown to hydrolyse a wide range of small molecule nitriles to the corresponding carboxamides and carboxylic acids [18].

The *Rhodococcus rhodochrous* nitrile hydratase is composed of α and β subunits and typically exists as a stable αβ heterodimer or α₂β₂ heterotetramer [19]. The metal ion (Co³⁺) at the catalytic centre is deeply buried within the α-subunit at the end of a channel lined with bulky aromatic amino acids that control access of substrates [20]. Navigation of small aliphatic nitriles through the channel of the Co-Nhase of *Pseudonocardia thermophila* has been simulated through computer modelling [21]. The channel and the active site configuration permit enantioselective hydration of nitriles [22,23,24], but are therefore likely to be highly constrained and limit the types of nitrile compounds that will be converted based on configuration and size [25].

Early studies revealed that *R. rhodochrous* J1 resting cells could catalyse the conversion of the aromatic nitriles benzonitrile, 2,6-difluorobenzonitrile, indoleacetonitrile, thiophenenitrile and furanenitrile to the corresponding amides [11]. A high-molecular-mass nitrile hydratase (H-NHase) was purified from this organism, and it is this enzyme that is responsible for the commercial production of acrylamide. Nagasawa et al. performed a broad comparative analysis of the H-NHase’s substrate profile using 61 small nitrile compounds [26]. This confirmed that the enzyme preferred short unbranched aliphatic nitriles, particularly acetonitrile (maximum activity), chloroacetonitrile, acrylonitrile and propionitrile. It demonstrated only a small fraction of that activity (less than 5%) against *n*-valeronitrile (pentanenitrile) and *n*-capronitrile (hexanenitrile) and no activity against the branched isovaleronitrile (3-methylbutanenitrile). The enzyme has lower activity against aromatic compounds and demonstrated only 17% activity (compared to acetonitrile) against 3-cyanopyridine and 11% against benzonitrile, with substitutions of benzonitrile further reducing conversion.

A second nitrile hydratase was found to be expressed by the same organism. Although also a Co-NHase and with relatively similar structure to the L-NHase, the substrate preference differed. The purified cobalt nitrile hydratase (named low molecular mass nitrile hydratase; L-NHase) of *R. rhodochrous* J1 catalysed the conversion of the aliphatic nitriles chloroacetonitrile, *n*-butyronitrile, propionitrile, methacrylonitrile, acrylonitrile and crotononitrile. Wieser et al. reported that L-NHase also exhibited a preference for branched chain aliphatic compounds (isobutyronitrile and methacrylonitrile) and aromatic nitriles (benzonitrile, 2-cyanopyridine, 3-cyanopyridine, 4-cyanopyridine, 2-cyanopyrazine) that was magnitudes higher compared to the H-NHase, as measured by the catalytic kinetic parameter Vmax/Km [27]. The L-NHase also had high catalytic activity for 2-, 3-, and 4-aminobenzonitrile. This therefore makes the L-NHase more interesting to synthetic chemists than the H-NHase [28].

The later in a synthetic sequence a compound can be transformed, the wider the possible retrosynthetic options. Hence, it is useful for organic synthetic chemists to have the option of transforming nitrile groups not just on small molecules but also on much larger compounds. For example, hydrolysis of nitrile groups of larger substrates such as the superficial nitrile groups of the polymer polyacrylonitrile (molecular mass, 190 kDa) has been demonstrated using *R. rhodochrous* NCIMB 11216 NHase [29]. Although nitrile hydratase activity has been evaluated on a wide range of nitrile substrates [30] a comprehensive evaluation of the NHase catalytic active site has not been performed. *R. rhodochrous* strain ATCC BAA-870 was found to express a Co-NHase with an amino acid sequence identical to that of the low molecular mass Co-NHase of *R. rhodochrous* J1 [31]. Herein, we report on the biocatalytic activity of *R. rhodochrous* strain ATCC BAA-870 NHase on a variety of nitriles of various sizes and substituents.

## 2. Results and Discussion

**Scheme 2:**
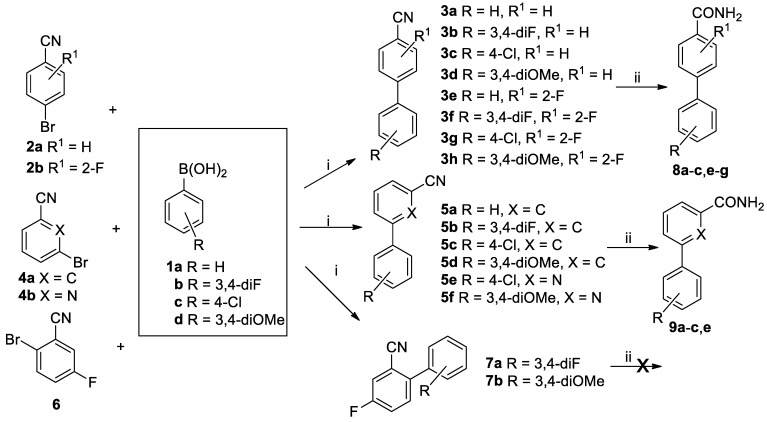
Suzuki reaction followed by biocatalysis reaction. Conditions: (**i**) Pd(PPh_3_)_4_, Na_2_CO_3_, DME; (**ii**) NHase, Tris buffer pH 7.6, acetone, 30 °C.

Apart from evaluating a range of readily available small aromatic nitriles, we synthesised a number of more functionalised derivatives for assessing the biocatalytic potential of the purified L-NHase produced by *R. rhodochrous* ATCC BAA-870. Biaryl substrates were prepared using the Suzuki coupling reaction (Scheme 2), where boronic acids **1a–d** were reacted with 4-bromobenzonitriles **2a–b**, to give the *para*-substituted biaryl derivatives **3a–h**. 3-Bromobenzonitrile **4a** and cyanopyridine **4b** were reacted similarly to give products **5a–f**, while 2-bromo-5-fluorobenzonitrile **6** gave *ortho*- substituted biaryl products **7a–b**.

Purified nitrile hydratase (NHase) was used in the biocatalysis reaction (step ii, Scheme 2) to avoid complications arising from background levels of amidase and nitrilase activity present in the cells (Scheme 1) and any filtering effect of the cell membrane. A number of *para*-substituted biaryl compounds **3** (see Supplementary Materials for spectroscopic data) were converted into their corresponding amide derivatives **8** by nitrile hydratase, as were compounds **5** into their corresponding amides **9**. However, neither of the two *ortho*-substituted biaryl compounds was converted in the biocatalytic reaction.

**Scheme 3:**
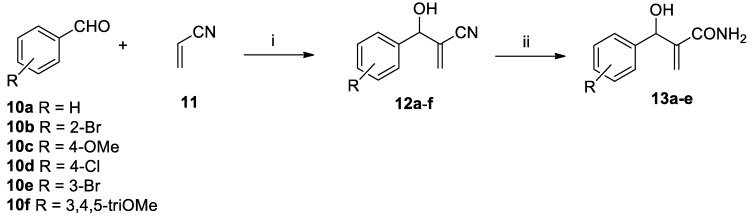
Morita Baylis–Hillman reaction and biocatalysis reaction. Conditions: (**i**) DABCO; (**ii**) NHase, Tris buffer pH = 7.6, acetone, 30 °C.

The DABCO-catalysed Morita–Baylis–Hillman (MBH) reaction (Scheme 3) between an aldehyde **10a–f** and acrylonitrile **11** gave MBH adducts **12a–f** in good yield. These were successfully converted into the corresponding amides **13a–e** using nitrile hydratase (step ii, Scheme 3). Compounds **12c**–**e** did not show full conversion, while **12f** was not converted at all.

**Scheme 4:**
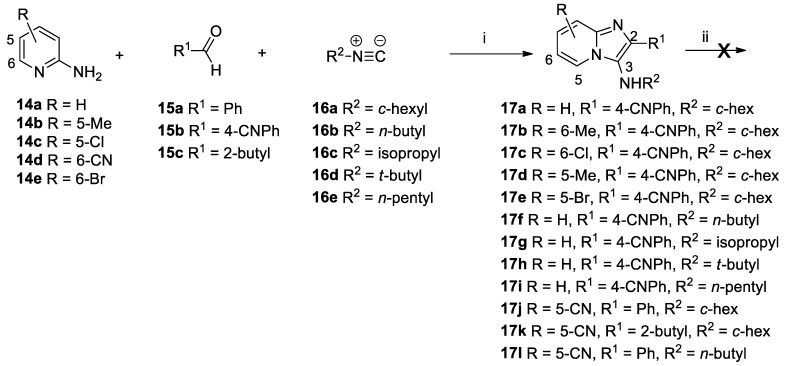
Groebke–Blackburn–Bienaymé multicomponent reaction, followed by biocatalysis reaction. Conditions: (**i**) Montmorillonite K-10 clay, dioxane, 100 °C; (**ii**) NHase, Tris buffer pH = 7.6, acetone, 30 °C.

The Groebke–Blackburn–Bienaymé multicomponent reaction between an aminopyridine **14a–e**, aliphatic or aromatic aldehyde **15a–c** and an isocyanide **16a–e** was used for the preparation of imidazo[1,2-*a*]pyridines **17a–l**, containing a nitrile functional group (Scheme 4). These were subjected to nitrile hydrolysis conditions using nitrile hydratase, but none of these derivatives was converted into the corresponding amide.

**Scheme 5:**
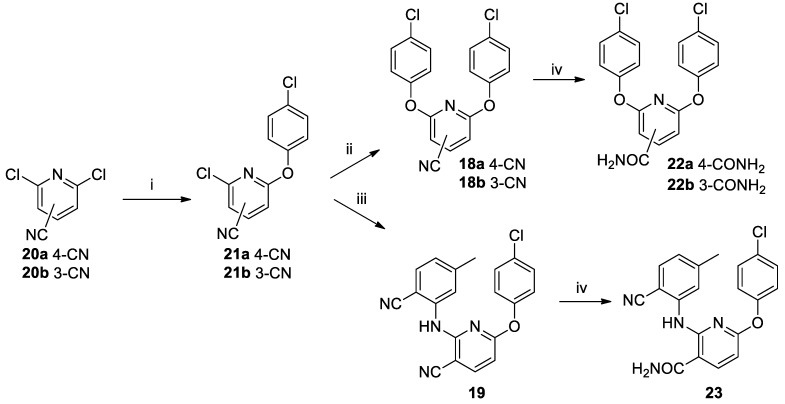
Buchwald–Hartwig reactions, followed by biocatalysis. Conditions: (**i**) and (**ii**) 4-chlorophenol, Pd_2_dba_3_, *rac*-BINAP, dioxane, NaO*t*Bu, 110 °C; (**iii**) 2-cyano-5-methylaniline, Pd_2_dba_3_, *rac*-BINAP, 1,4-dioxane, NaO*t*Bu, 110 °C; (**iv**) NHase, Tris buffer pH = 7.6, acetone, 30 °C.

Finally, three cyano-substituted pyridyl derivatives **18a**–**b** and **19** bearing two aromatic rings linked through heteroatoms were prepared and evaluated as possible substrates for nitrile hydratase. The compounds were prepared according to the route shown in Scheme 5. Dichloropyridyl derivatives **20a**–**b** were reacted under Buchwald–Hartwig cross-coupling conditions to give 4-chlorophenyl-substituted derivatives **21a**–**b**. These compounds were reacted under similar conditions to give the *bis*-4-chlorophenyl derivatives **18a**–**b**. Reaction of **21b** with 2-cyano-5-methylaniline gave compound **19**. Subjecting compounds **18a**–**b** to nitrile hydratase in buffer gave rise to amides **22a**–**b**. The same reaction conditions for compound **19**, containing 2 nitrile groups, gave rise exclusively to compound **23**, where the nitrile on the pyridine ring had been converted to the corresponding amide, leaving the nitrile on the benzene ring unreacted.

**Figure 1:**
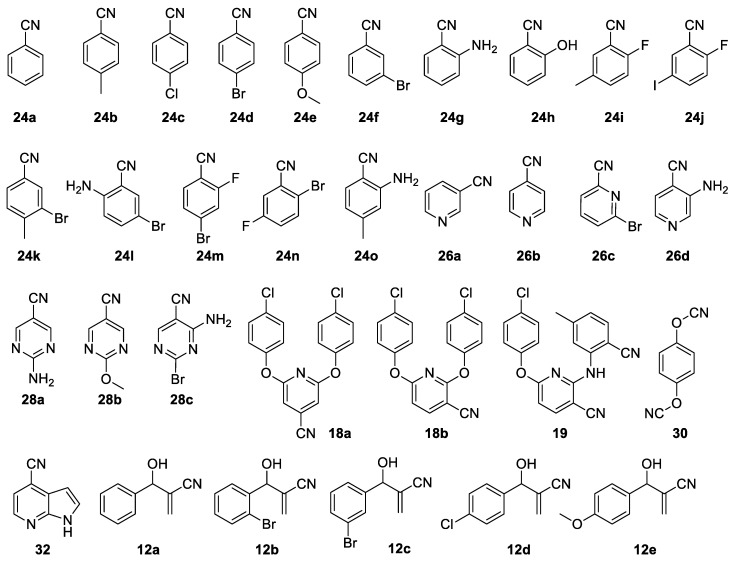
Compounds showing 50% to 100% conversion.

**Figure 2:**
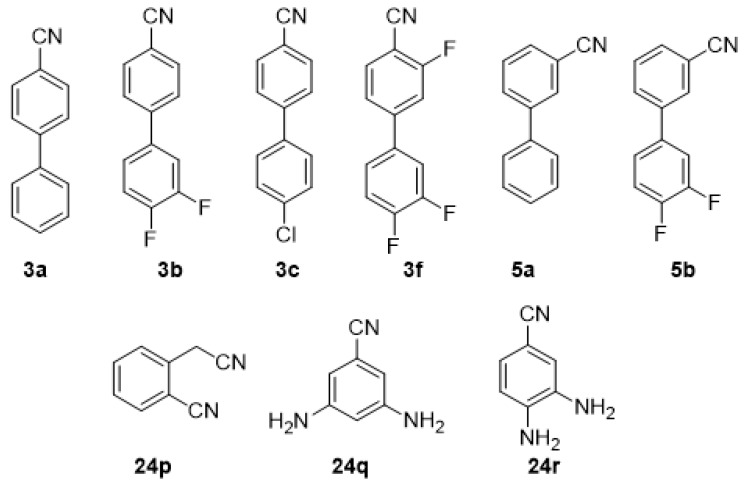
Compounds showing 16% to 50% conversion.

**Figure 3:**
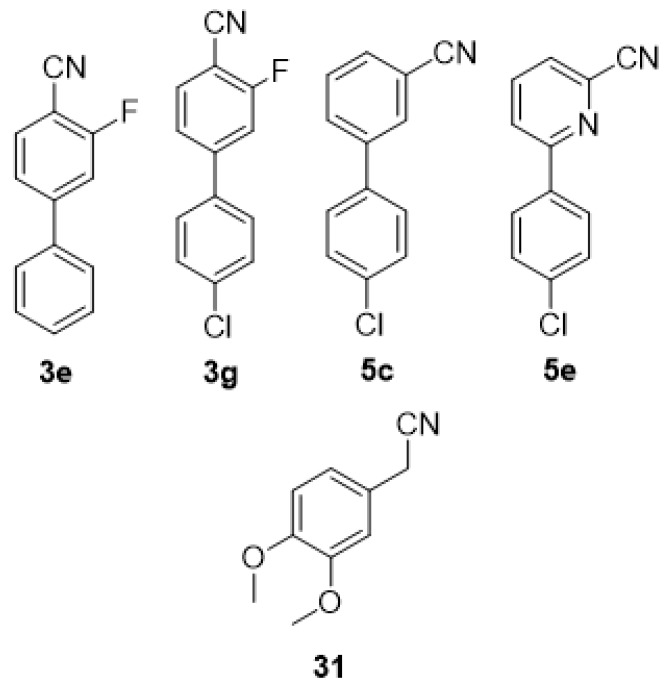
Compounds showing 5% to 15% conversion.

**Figure 4:**
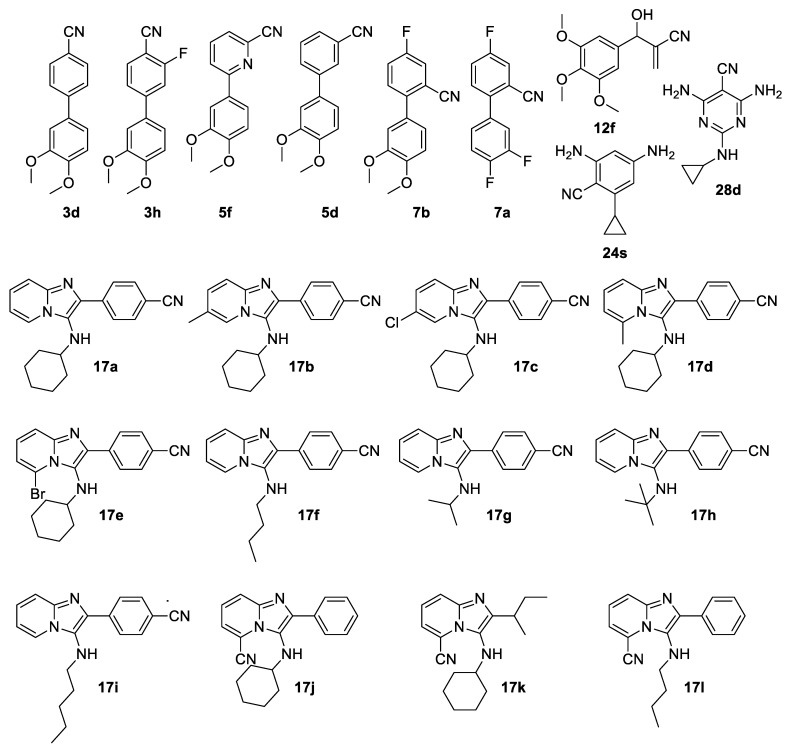
Compounds showing 0% to 5% conversion.

All the substrates evaluated are presented in Figure 1, Figure 2, Figure 3 and Figure 4, and they have been grouped according to their extent of conversion. Thus, Figure 1 shows compounds for which conversions of 50% to 100% were observed, Figure 2 shows compounds with 16% to 50% conversion, Figure 3 shows compounds that were converted between 5% and 15% and Figure 4 shows compounds that were essentially not converted. The small substrate compounds include benzonitrile derivatives **24a**–**s** that were converted to the corresponding amides **25a**–**r**, cyanopyridines **26a**–**d** converted to the amides **27a**–**d** and cyanopyrimidines **28a**–**d** converted to the amides **29a**–**c**. In addition, two phenol derivatives (**30** and **31**) and an azaindole (**32**) were also evaluated. Compound **30** was converted into the mono-amide product **33**, while compounds **31** and **32** were converted to the corresponding amides **34** and **35**, respectively. Evidently, the NHase of *R. rhodochrous* ATCC BAA-870 readily converted most of these small aromatic nitriles (Figure 1).

There were two main factors that appeared to contribute to the absolute ability of NHase to hydrolyse a substrate to the corresponding amide and to contribute to the rate at which conversion occurred: steric hindrance and electronic properties of the nitrile. For example, two highly sterically hindered compounds, benzonitrile derivative **24s** and a pyrimidine derivative **28d**, bearing a substituent on either side the nitrile group, were the only small molecules not converted to the corresponding amides (Figure 4). A single large substituent adjacent to the nitrile group, however, had no negative influence on conversion to the corresponding amide by NHase. For example, 2-bromo-5-fluorobenzonitrile (**24n**) was fully converted within 24 h into the amide **25n**. This indicates that significant steric hindrance is required immediately in the vicinity of the nitrile to affect the capacity of NHase to hydrolyse these small aromatic substrates. This finding is supported by earlier work where steric hindrance caused problems with conversion of 2,6-disubstituted benzonitriles for a purified NHase from a strain of *Rhodopseudomonas palustris*. In this study, 2,6-difluorobenzonitrile was readily converted, while replacement of the F atoms with the larger chlorine atoms in 2,6-dichlorobenzonitrile (the herbicide dichlobenil) reduced conversion to a negligible level [32]. This clearly points to steric hindrance rather than electronic effects being responsible for the change in reactivity of the two substrates. Interestingly, whole cells of *R. rhodochrous* PA-34 have proved to be somewhat effective in converting dichlobenil, generating 48% amide after 3 d. [33].

The cyano group of nitriles is very polar, permitting high reactivity through the electrophilicity of the nitrile carbon atom. However, this electrophilicity is moderated by the electronic properties of neighbouring atoms and the electron density of the aromatic rings to which they are attached. If electrophilicity of the nitrile carbon atom were a significant factor in determining conversion by NHase, it would be expected that substrates with electron-releasing groups attached to the aromatic ring would not be converted by the enzyme or would be converted more slowly, while substrates with electron-withdrawing groups would show increased activity. Within the group of benzonitriles tested, it does indeed appear that benzonitriles bearing electron-donating groups on the aromatic ring were converted at a slower rate than those bearing electron-withdrawing groups.

For example, benzonitrile (**24a**), 3-cyanopyridine (**26a**) and 4-cyanopyridine (**26b**) were all fully converted to the corresponding amide after 24 h. However, 4-methoxybenzonitrile (**24e**) and 4-methylbenzonitrile (**24b**) with electron-donating substituents took 48 h for full conversion to the corresponding amides. Conversely, compounds such as 4-bromobenzonitrile (**24d**) and 2-fluoro-4-bromobenzonitrile (**24m**) with electron-withdrawing groups were fully converted after 24 h. Thus, the electrophilicity of the carbon atom of the nitrile appears to alter the rate of conversion but not the absolute ability of NHase to hydrolyse a particular substrate.

Of interest is the fact that *ortho*-substituted benzonitriles bearing an electron-donating group such as the 2-aminobenzonitrile (**24g**) and 2-hydroxybenzonitrile (**24h**) were still fully converted but also at a slower rate, taking 48 h for conversion. Thus, although steric hindrance could have played a role for these substrates, it seems that electronic properties dominated here, and slowed down the rate of conversion. This is supported by the result of 2-amino-5-bromobenzonitrile (**24l**), which was converted fully within 24 h. Thus, the presence of the electron-withdrawing bromo-group *para* to the amino group changes the electronic properties of the attached nitrile, making it more reactive and thus readily converted to the corresponding amide at a faster rate than the derivative lacking the bromo-substituent (**24g**). The results are in broad agreement with those of Wieser et al. [27], who found that, unlike the H-NHase profiled by Nagasawa et al. [26], the L-NHase readily accepts *ortho*-substituted benzonitriles such as 2-hydroxybenzonitrile, 2-nitrobenzonitrile, 2-aminobenzonitrile and 2-methylbenzonitrile.

**Figure 5:**
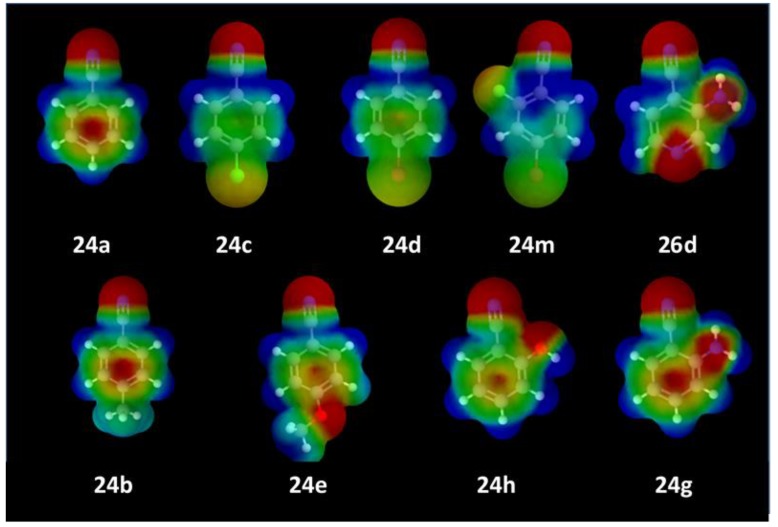
MEP diagrams. Blue indicates electron deficient and red electron rich areas, respectively.

A comparison of the results for 2-aminobenzonitrile (**24g**) and the pyridyl equivalent (**26d)** shows that the presence of the nitrogen in the ring enhances reaction rate, with **24g** taking 48 h to reach full conversion and **26d** being converted fully within 24 h. A similar result was seen for pyrimidine derivative **28a**, where full conversion was achieved within 24 h. This is not unexpected as the ring nitrogen atoms decrease the electron density of the ring, thus increasing the electrophilicity of the nitrile carbon atom. The enzyme activity is sensitive to the electron density of the aromatic ring, which influences the electrophilicity of the nitrile carbon atom. Figure 5 clearly shows the difference in electron density around the nitrile carbon atom for compounds **24a**, **24c**, **24d**, **24m**, **26d,** which were converted in 24 h, and compounds **24b**, **24e**, **24h**, **24g,** which were converted only after 48 h. A comparison of **26d** and **24g** illustrates how introduction of a nitrogen atom into the ring decreases the electron density around the nitrile carbon atom, with a corresponding increase in reactivity (Figure 5).

**Figure 6:**
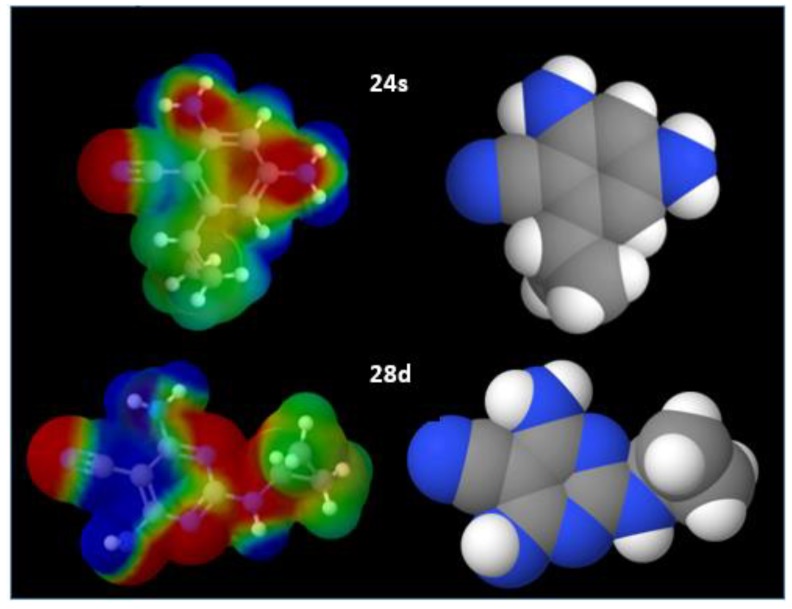
Compounds **24s** and **28d** MEP and space filling models.

Where two large groups are found adjacent to the nitrile, steric hindrance is clearly the dominant factor, leading to these substrates not being converted into the corresponding amides. Figure 6 shows the MEP and space-filling models for compounds **24s** and **28d**, which clearly show the significant difference in electron density around the nitrile carbon atom between the two compounds, with that of the pyrimidine **28d** being considerably more electron-deficient than that for **24s**. Despite the expected high reactivity of **28d** due to the electrophilicity of the nitrile carbon if electronic factors were dominant, no reaction was observed. This points strongly to steric hindrance, rather than electronic properties, being the more significant factor for conversion of a substrate by NHase in the case of aromatic nitriles bearing two adjacent substituents, as discussed earlier.

Although the *para*-substituted biaryl compounds **3** were accepted by the enzyme, their incomplete conversion (Figure 2) perhaps indicates that their bulky nature makes them less desirable as substrates. The 3′,4′-dimethoxy substituted compounds **3d** and **3h** were an exception, as they were not converted to any extent (Figure 4). Methoxy groups tend to be electron donors, and hence may reduce the electrophilicity of the nitrile. However, even when present on the same aromatic ring as the nitrile, this effect appears moderate, and one can expect that it would be reduced when on the second ring of a biaryl system, so it is more likely that the effect here is steric in nature. Fewer of the biaryl systems with the second ring *meta* to the nitrile (**5**) were converted (Figure 2 and Figure 3), and again, the 3′,4′-dimethoxy substituted compounds (**5d** and **5f**) were not converted (Figure 4). The two compounds with aromatic rings *ortho* to the nitrile (**7a–b**) were not converted at all (Figure 4), presumably due to steric hindrance preventing access to the cyano group or an inability to orientate correctly in the active site.

The Morita–Baylis–Hillman products allowed exploration of reactivity of a constrained nitrile group attached to a non-aromatic sp^2^ carbon atom. The parent compound (**12a**) and the 2-bromosubstituted adduct (**12b**) were completely converted within 48 h to the corresponding amides (Figure 1). The two *para*-substituted adducts (choro (**12d**) and methoxy (**12e**)) and the bromo *meta*-substituted adduct (**12c**) were not fully converted even after 5 d (Figure 2), but all three still gave reasonable isolated yields of amide of above 50%. This is a synthetically useful transformation, as direct synthesis of amides using the Morita–Baylis–Hillman reaction with acrylamide is not possible [34]. The only substrate not accepted by the enzyme was the tri-methoxy substituted adduct (**12f**, Figure 4) and its complete lack of conversion suggests steric constraints related to the enzyme active site channel, with two bulky substituents positioned *meta* to the aliphatic group. Enzymatic conversion of the nitrile group of Morita–Baylis–Hillman adducts has previously been demonstrated using whole cells of *Rhodococcus* sp. AJ270, where a mixture of carboxamides and carboxylic acids was obtained, presumably as a result of the presence of both NHase and amidase [35].

**Figure 7:**
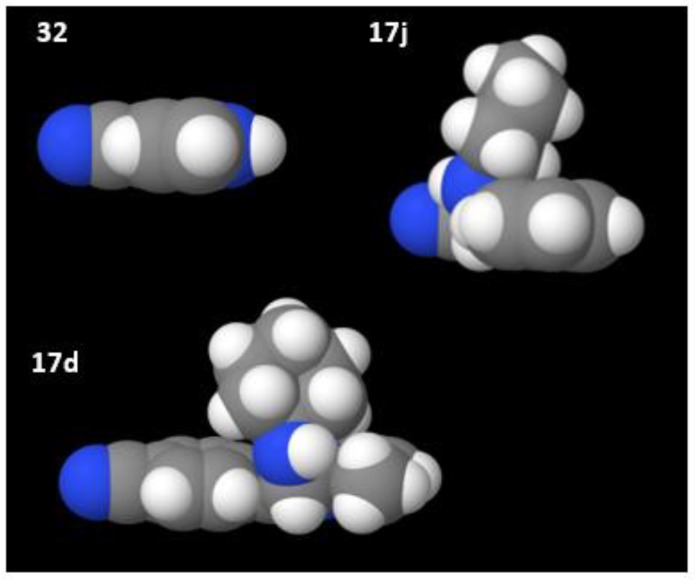
Space filling models of 7-azaindole-4-carbonitrile **32**, and Groebke–Blackburn–Bienaymé multicomponent reaction products **17j** and **17d.**

The simple 6-5-ring-fused heterocycle 7-azaindole-4-carbonitrile (**32**) was completely converted (Figure 1). However, none of the substituted 6-5-ring fused imidazo[1,2-*a*]pyridin-3-amine derivatives generated by the Groebke–Blackburn–Bienaymé multicomponent reaction (**17a–l**) were converted, in spite of the cyano group being placed in different positions (Figure 4). The space-filling models (van der Waals spheres) of these compounds show that they are considerably more bulky and therefore less likely to access the active site than the simple aza-indole **32** (Figure 7).

The channel to the enzyme active site is about 7 Å deep, at least, in the case of Co-NHase of *Pseudonocardia thermophila* JCM 3095, and steered molecular dynamics computer modelling revealed that the entrance to the active site may adopt a much larger size than that determined for the rigid X-ray structure. [21,36]). In the current study, substrate length was not a limiting factor, with compound **18a** being a readily converted substrate. However, a diameter beyond about 5 Å could not be accommodated, as seen for substrates **17**. A factor contributing to the successful conversion of the large compounds **18a**, **18b** and **19** to the corresponding amides (Figure 1) may be their linkage through heteroatoms. This permits a high level of torsional flexibility, allowing them to adopt multiple conformations, thus enabling them to access the active site.

**Figure 8:**
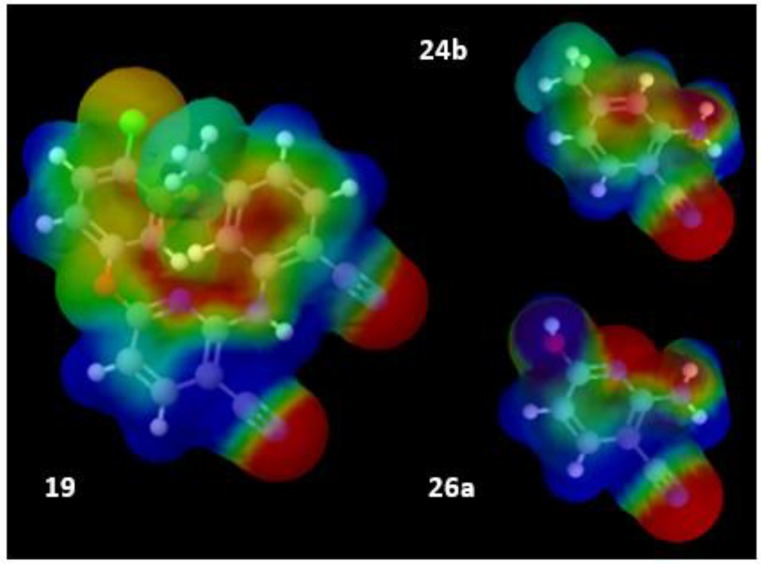
MEP of **19** with comparison to 3-cyanopyridine (**26a**), and 4-methylbenzonitrile (**24b**).

Regio-selective hydration of dinitriles is a great advantage of biocatalysis. There are numerous reports of selective hydration of one nitrile group in dinitriles using whole cells with nitrile hydratase activity [37,38,39]. This is explained in part by kinetics—the mono-nitrile only becomes available as the reaction proceeds, while there is double the likelihood of converting a compound that bears two cyano groups. Steric hindrance may also exert an effect, for example in compound **24p**, which was converted into **25p**, only the aliphatic nitrile was converted to the amide, leaving the aromatic nitrile unreacted. This may be a result of the pendant nitrile group being more readily accessible. Electronic effects may also arise from the contribution of the new amide substituent that is less electron-withdrawing than the original nitrile, for example in the conversion of **30** to **33**. In the case of compound **19** the cyano groups experience non-identical electron densities, one similar to 3-cyanopyridine (**26a**) and one similar to the more slowly converted 4-methylbenzonitrile (**24b**) (Figure 8). The nitrile of the cyanopyridine is preferentially converted. Lastly, the active site can also control the degree of specificity, with Cheng et al. [40] demonstrating that modification of the active site can improve absolute regiospecificity in the L-Nhase.

## 3. Conclusion

We have comprehensively explored the substrate parameters for the J1 type low molecular mass cobalt nitrile hydratase in the hydration of a wide range of aromatic nitriles and aliphatic nitriles containing an aromatic ring. The enzyme accepted all but three of the small nitrile compounds tested, indicating a relatively accommodating active site. Moreover, the electrophilicity of the nitrile seems to have a limited influence on activity, slowing down but not preventing reactions where the nitrile carbon atom is less electrophilic. This could perhaps be due to the capacity of the active site to minimise this effect on the reaction by increasing substrate electrophilicity in situ. Steric hindrance, for example, by large vicinal substituents present on both sides of the nitrile did prevent conversion. Lastly, some substrates appear to have been too large and rigid to be accommodated by the enzyme’s catalytic site or the channel leading to it. Recent research indicates that this may be overcome by enzyme engineering. The next logical steps in the development of this enzyme are a combination of enzyme modelling, substrate docking of the molecules tested here to prove the model and subsequent protein engineering to determine how the substrate profile can be expanded.

## 4. Materials and Methods

General Chemicals were purchased from Sigma-Aldrich (Darmstadt, Germany) and were used without further purification. Thin layer chromatography (TLC) was performed on aluminium-backed Merck silica gel 60 F_254_ plates (Darmstadt, Germany). Column chromatography was performed using gravity (particle size 0.063–0.200 mm) or flash (particle size 0.040–0.063 mm) silica gel 60 purchased from Merck (Darmstadt, Germany). ^1^H and ^13^C NMR spectra were recorded using a Bruker AVANCE 300, 400 or 500 MHz spectrometer (Bruker, Mass., USA). The biocatalytic reactions were conducted at small scale and followed by TLC to monitor conversion to the corresponding amide. Where the spot corresponding to the starting material disappeared completely, this was noted as full conversion for the purposes of the figures and discussion in the paper. However, in the experimental section the isolated yield is recorded. Due to the differences in ease of isolation of the different products, for a few substrates there was a significant discrepancy between conversion and isolated yield.

Suzuki coupling reaction: general procedure. A benzonitrile derivative was reacted with the boronic acid (1.2 eq) in the presence of Pd(PPh₃)₄ (5 mol%) and 2M Na₂CO₃ in DME, and the reaction mixture was refluxed overnight under a nitrogen atmosphere. After completion, the reaction was cooled and ethyl acetate and water were added. After separation, the organic layer was dried over Na_2_SO_4_ and the solvent removed under reduced pressure. The mixture was then purified using flash silica gel column chromatography using 5% ethyl acetate/hexane.

The Morita–Baylis–Hillman reaction: general procedure. A mixture of aldehyde (0.098 mol), acrylonitrile (40 mL, 0.608 mol) and DABCO (10.9 g, 0.098 mol) was stirred at 0 °C for 19 h. After completion of the reaction, ethyl acetate and water were added to the reaction mixture. The organic layer was separated, dried over MgSO_4_, and the solvent was removed in vacuo. Products were purified by silica gel column chromatography, eluting with ethyl acetate/hexane.

Groebke–Blackburn–Bienaymé multicomponent reaction: general procedure. 2-Aminopyridine (1.33 mmol), aldehyde (1.33 mmol), isocyanide (1.36 mmol) and montmorillonite K-10 clay (250 mg) were reacted in dioxane overnight at 100 °C. After reaction, water and ethyl acetate were added, and after extraction, the organic layer was concentrated in vacuo. The desired product was purified by silica gel flash chromatography, eluting with ethyl acetate and hexane.

General method for preparation of **18a** and **18b**. Tris-(dibenzylideneacetone)-di-palladium (0) [Pd_2_dba_3_] (28 mg, 3 mol %), *rac*-BINAP (38 mg, 6 mol %), 1,4-dioxane (3–5 mL) and a magnetic stirrer (Sigma-Aldrich, Darmstadt, Germany)) were added to an oven-dried 10 mL round bottomed flask and purged with nitrogen. The flask was sealed and heated with stirring at 80 °C in an oil bath for 5 min. Thereafter, the appropriate carbonitrile substrate (1.0 mmol), 4-chlorophenol (128 mg, 2.0 mmol)) and sodium *tert*-butoxide (1.5 mmol) were added and the sealed reaction heated at 110–120 °C for 24 h. The cooled reaction mixture was filtered, and the excess solvent removed in vacuo to leave a crude mixture which was re-dissolved in dichloromethane and filtered. The filtrate was washed successively with aqueous saturated NaHCO_3_ (10 mL) and distilled water (2 × 10 mL). After drying over anhydrous Na_2_SO_4_ and evaporating excess solvent, the crude mixture was purified by silica gel flash column chromatography, eluting the target with 0% to 3% EtOAc/hexane.

Biocatalysis reaction: general procedure. The reaction was carried out using a 1:1 mass ratio of purified NHase [41] and the substrate, with a total reaction volume of 2 mL.

Composition of the reaction mixture: 1800 μL (90%) Tris buffer (50 mM, pH 7.6) and 200 μL (10%) of methanol or acetone. In a 2 mL Eppendorf, NHase (10 mg) was added followed by Tris buffer. Nitrile substrate (10 mg dissolved in 200 μL methanol or acetone) was added to the 2 mL Eppendorf tube. (If an amine group was present on the nitrile substrate a Tris buffer of pH 9 was used). The reaction mixture was incubated at 30 °C on an ESCO Provocell microplate shaker/incubator (Esco Technologies, Halfway House, South Africa) (199 rpm). The reaction was allowed to proceed for 24 h, 48 h or 5 d, depending on conversion, as monitored by TLC analysis. Ethyl acetate and water were added to the reaction mixture, and after separation, the organic layer was concentrated under reduced pressure, and the resulting mixture was then purified by silica gel column chromatography eluting with 20% to 90% ethyl acetate/hexane.

## Supplementary Materials

The following are available online, spectroscopic data for compounds **3**, **5**, **7**–**9**, **12**, **13**, **17**–**19**, **22**, **23**, **25**.

## References

[1] Parkins A.W. (1996). Catalytic hydration of nitriles to amides. Platinum-containing catalyst offers new opportunity. Platinum Metals Rev..

[2] Nagasawa T., Shimizu H., Yamada H. (1993). The superiority of the third-generation catalyst, Rhodococcus rhodochrous J1 nitrile hydratase, for industrial production of acrylamide. Appl. Microbiol. Biotechnol..

[3] Yamada H., Kobayashi M. (1996). Nitrile hydratase and its application to industrial production of acrylamide, Biosci. Biotechnol. Biochem..

[4] Li B., Su J., Tao J. (2011). Enzyme and Process Development for Production of Nicotinamide. Org. Proc. Res. Dev..

[5] Hann E.C., Eisenberg A., Fager S.K., Perkins N.E., Gallagher F.G., Cooper S.M., Gavagan J.E., Stieglitz B., Hennessey S.M., DiCosimo R. (1999). 5-Cyanovaleramide production using immobilized Pseudomonas chlororaphis B23. Bioorg Med Chem..

[6] Gong J.S., Shi Z.M., Lu J.S., Li H., Zhou Z.M., Xu Z.H. (2017). Nitrile-converting enzymes as a tool to improve biocatalysis in organic synthesis: Recent insights and promises. Crit. Rev. Biotechnol..

[7] Lanfranchi E., Steiner K., Glieder A., Hajnal I., Sheldon R.A., van Pelt S., Winkler M. (2013). Mini-Review: Recent developments in hydroxynitrile lyases for industrial biotechnology. Recent Pat. Biotechnol..

[8] van Rantwijk F., Stolz A. (2015). Enzymatic cascade synthesis of (*S*)-2-hydroxycarboxylic amides and acids: Cascade reactions employing a hydroxynitrile lyase, nitrile-converting enzymes and an amidase. J. Mol. Cat. B: Enz..

[9] Bhalla T.C., Kumar V., Kumar V., Thakur N. (2018). Nitrile Metabolizing Enzymes in Biocatalysis and Biotransformation. Appl. Biochem. Biotechnol..

[10] Brennan B.A., Alms G., Nelson M.J., Durney L.T., Scarrow R.C. (1996). Nitrile Hydratase from Rhodococcus rhodochrous J1 contains a non-corrin cobalt ion with two sulphur ligands. J. Am. Chem. Soc..

[11] Mauger J., Nagasawa T., Yamada H. (1989). Synthesis of various aromatic amide derivatives using nitrile hydratase of Rhodococcus rhodochrous J1. Tetrahedron.

[12] Kobayashi M., Komeda H., Nagasawa T., Yamada H., Shimizu S. (1993). Occurrence of amidases in the industrial microbe Rhodococcus rhodochrous J1, Biosci. Biotechnol. Biochem..

[13] Beard T.M., Page M.I. (1998). Enantioselective biotransformations using rhodococci. Antonie van Leeuwenhoek..

[14] Kobayashi M., Nagasawa T., Yamada H. (1989). Nitrilase of Rhodococcus rhodochrous J1. Purification and characterization. Eur. J. Biochem..

[15] Meth-Cohn O., Wang M.X. (1997). An in-depth study of the biotransformation of nitriles into amides and/or acids using Rhodococcus rhodochrous AJ270. J. Chem. Soc. Perkin Trans..

[16] Klempier N., Harter G., de Raadt A., Griengl H., Braunegg G. (1996). Chemoselective hydrolysis of nitriles by Rhodococcus rhodochrous NCIMB 11216. Food Technol. Biotechnol..

[17] Fernandes B.C.M., Mateo C., Kiziak C., Chmura A., Wacker J., van Rantwijk F., Stolz A., Sheldon R.A. (2006). Nitrile hydratase activity of a recombinant nitrilase. Adv. Synth. Catal..

[18] Martínková L., Mylerová V. (2003). Synthetic applications of nitrile-converting enzymes. Curr. Org. Chem..

[19] Hashimoto Y., Sasaki S., Herai S., Oinuma K., Shimizu S., Kobayashi M. (2002). Site-directed mutagenesis for cysteine residues of cobalt-containing nitrile hydratase. J. Inorg. Biochem..

[20] Pravda L., Berka K., Svobodová Vařeková R., Sehnal D., Banáš P., Laskowski R.A., Koča J., Otyepka M. (2014). Anatomy of enzyme channels. BMC Bioinformatics.

[21] Peplowski L., Kubiak K., Nowak W. (2008). Mechanical aspects of nitrile hydratase enzymatic activity. Steered molecular dynamics simulations of Pseudonocardia thermophila JCM 3095. Chem. Phys. Lett..

[22] Kinfe H.H., Chhiba V., Frederick J., Bode M.L., Mathiba K., Steenkamp P.A., Brady D. (2009). Enantioselective hydrolysis of β-hydroxy nitriles using the whole cell biocatalyst of Rhodococcus rhodochrous ATCC BAA-870. J. Mol. Catal. B Enz..

[23] Wang M.X., Li J.J., Ji G.J., Li J.S. (2001). Enantioselective biotransformations of racemic 2-aryl-3-methylbutyronitriles using Rhodococcus sp. AJ270. J. Mol. Catal. B: Enz..

[24] Chhiba V.P., Bode M.L., Mathiba K., Kwezi W., Brady D. (2012). Enantiomeric biocatalytic hydrolysis of β-aminonitriles to β-aminoamides using Rhodococcus rhodochrous ATCC BAA-870. J. Mol. Catal. B Enz..

[25] Peplowski L., Kubiak K., Nowak W. (2007). Insights into catalytic activity of industrial enzyme Co-nitrile hydratase. Docking studies of nitriles and amides. J. Mol. Modeling.

[26] Nagasawa T., Takeuchi K., Yamada H. (1991). Characterization of a new cobalt-containing nitrile hydratase purified from urea-induced cells of Rhodococcus rhodochrous J1. Euro. J. Biochem..

[27] Wieser M., Takeuchi K., Wada Y., Yamada H., Nagasawa T. (1998). Low-molecular-mass nitrile hydratase from Rhodococcus rhodochrous J1: Purification, substrate specificity and comparison with the analogous high-molecular-mass enzyme. FEMS Microbiol. Lett..

[28] Mauger J., Nagasawa T., Yamada H. (1988). Nitrile hydratase-catalyzed production of isonicotinamide, picolinamide and pyrazinamide from 4-cyanopyridine, 2-cyanopyridine and cyanopyrazine in Rhodococcus rhodochrous J1. J. Biotechnol..

[29] Tauber M.M., Cavaco-Paulo A., Robra K.H., Gübitz G.M. (2000). Nitrile hydratase and amidase from Rhodococcus rhodochrous hydrolyze acrylic fibers and granular polyacrylonitriles. Appl. Environ. Microbiol..

[30] MartÍnková L., Křen V. (2002). Nitrile- and amide-converting microbial enzymes: Stereo-, regio- and chemoselectivity. Biocat. Biotrans..

[31] Komeda H., Kobayashi M., Shimizu S. (1996). A novel gene cluster including the Rhodococcus rhodochrous J1 nhlBA genes encoding a low molecular mass nitrile hydratase (L-NHase) induced by its reaction product. J. Biol. Chem..

[32] Black G.W., Gregson T., McPake C.B., Perry J.J., Zhang M. (2010). Biotransformation of nitriles using the solvent-tolerant nitrile hydratase from Rhodopseudomonas palustris CGA009. Tetrahedron Lett..

[33] Veselá A.B., Pelantová H., Sulc M., Macková M., Lovecká P., Thimová M., Pasquarelli F., Pičmanová M., Pátek M., Bhalla T.C. (2012). Biotransformation of benzonitrile herbicides via the nitrile hydratase-amidase pathway in rhodococci. J. Ind. Microbiol. Biotechnol..

[34] Kim E.S., Lee H.S., Kim J.N. (2009). An efficient synthesis of Baylis–Hillman adducts of acrylamide: Pd-catalyzed hydration of Baylis–Hillman adducts of acrylonitrile. Tetrahedron Lett..

[35] Wang M.X., Wu Y. (2003). Nitrile biotransformations for the synthesis of enantiomerically enriched Baylis-Hillman adducts. Org. Biomol. Chem..

[36] Taştan Bishop A.O., Sewell T. (2006). A new approach to possible substrate binding mechanisms for nitrile hydratase Biochem. Biophys. Res. Comm..

[37] Meth-Cohn O., Wang M.X. (1997). Rationalization of the regioselective hydrolysis of aliphatic dinitriles with Rhodococcus rhodochrous AJ270. Chem. Commun..

[38] Dadd M.R., Timothy R., Claridge D.W., Walton R., Pettman A.J., Knowles C.J. (2001). Regioselective biotransformation of the dinitrile compounds 2-, 3- and 4-(cyanomethyl) benzonitrile by the soil bacterium Rhodococcus rhodochrous LL100–21. Enz. Microb. Technol..

[39] Brady D., Beeton A., Kgaje C., Zeevaart J., van Rantwijk F., Sheldon R.A. (2004). Characterisation of nitrilase and nitrile hydratase biocatalytic systems. Appl. Microbiol. Biotechnol..

[40] Cheng Z., Cui W., Xia Y., Peplowski L., Kobayashi M., Zhou Z. (2018). Modulation of nitrile hydratase regioselectivity towards dinitriles by tailoring the substrate binding pocket residues. ChemCatChem.

[41] Frederick J. Characterisation of the nitrile biocatalytic activity of Rhodococcus rhodochrous ATCC BAA-870. MSc Dissertation, University of the Witwatersrand. http://hdl.handle.net/10204/2872.

