# Supplementary material for: Substrate Profiling of the Cobalt Nitrile Hydratase from Rhodococcus rhodochrous ATCC BAA 870
Source: Molecules. 2020 Jan 6;25(1):238. doi: 10.3390/molecules25010238 (PMC6983157; doi:10.3390/molecules25010238)
Supplement: Supplementary file 1 [file molecules-25-00238-s001.pdf]

Supplementary information

Substrate Profiling of the Cobalt Nitrile Hydratase from *Rhodococcus rhodochrous* ATCC BAA 870

Adelaide. R. Mashweu [1], Varsha P. Chhiba-Govindjee [1,2], Moira L. Bode [1,*,†] and Dean Brady [1,*,†]

1 Molecular Sciences Institute, School of Chemistry, University of the Witwatersrand, Johannesburg 2050, South Africa; 1715870@students.wits.ac.za (A.R.M.); VChhiba@csir.co.za (V.P.C.-G.)

2 CSIR Chemical Production Cluster, PO Box 395, Pretoria 0001, South Africa

* Correspondence: Moira.Bode@wits.ac.za (M.L.B.); dean.brady@wits.ac.za (D.B.); Tel.: +27-117176745 (D.B.)

† These two authors contributed equally.

*Spectroscopic data for compounds **3**, **5** and **7***

[1,1'-Biphenyl]-4-carbonitrile (**3a**) [1] Yield 98% (white powder); mp 85-86°C; ^{1}H NMR (400 MHz, $CDCl_3$) δ 7.71 (d, *J* = 8.4 Hz, 2H), 7.67 (d, *J* = 8.3 Hz, 2H), 7.58 (d, *J* = 7.1 Hz), 7.48 (t, *J* = 7.3 Hz, 2H), 7.42 (t, *J* = 7.2 Hz, 1H); ^{13}C NMR (101 MHz, $CDCl_3$): δ 145.6, 139.1, 132.6, 129.1, 128.7, 127.7, 127.2, 118.9, 110.9.

3',4'-Difluoro-[1,1'-biphenyl]-4-carbonitrile (**3b**) [2]. Yield 98% (white powder); mp 105-106°C; ^{1}H NMR (400 MHz, $CDCl_3$): δ 7.76-7.71 (m, 2H), 7.65-7.60 (m, 2H), 7.40 (ddd, *J* = 11.2, 7.4, 2.2 Hz, 1H), 7.36-7.29 (m, 1H), 7.26-7.29 (m, 1H); ^{13}C NMR (101 MHz, $CDCl_3$): δ 150.8 (d, J_{CF} = 250.5, 12.6 Hz), 150.7 (d, J_{CF} = 251.5, 12.5 Hz), 143.5 (d, J_{CF} = 1.7 Hz), 136.3 (dd, J_{CF} = 5.9, 3.9 Hz), 132.8, 127.6, 123.4 (dd, J_{CF} = 6.4, 3.6 Hz), 118.6, 118.1 (d, J_{CF} = 17.5 Hz), 116.3 (d, J_{CF} = 18.1 Hz), 111.6.

4'-Chloro-[1,1'-biphenyl]-4-carbonitrile (**3c**) [3]. Yield 77% (white powder); mp 129-132°C; ^{1}H NMR (300 MHz, $CDCl_3$): δ 7.76-7.70 (m, 2H), 7.68-7.62 (m, 2H), 7.55-7.49 (m, 2H), 7.48-7.43 (m, 2H, H 9 & 13); ^{13}C NMR (75 MHz, $CDCl_3$): δ 144.7, 137.9, 135.3, 133.1, 129.7, 128.8, 127.9, 119.1, 111.6.

3',4'-Dimethoxy-[1,1'-biphenyl]-4-carbonitrile (**3d**) [1]. Yield 55% (yellow powder); mp 139-140°C; ^{1}H NMR (300 MHz, $CDCl_3$): δ 7.73-7.62 (m, 4H), 7.17 (dd, *J* = 8.3, 2.1 Hz, 1H), 7.09 (d, *J* = 2.0 Hz, 1H), 6.97 (d, *J* = 8.3 Hz, 1H), 3.96 (s, 3H), 3.94 (s, 3H); ^{13}C NMR (75 MHz, $CDCl_3$): δ 149.8, 149.5, 145.4, 132.7, 131.9, 127.3, 119.9, 119.0, 111.6, 110.3, 56.1, 56.0.

3-fluoro-[1,1'-biphenyl]-4-carbonitrile (**3e**).[4] Yield 82% (yellow powder); mp 102-104°C; ^{1}H NMR (400 MHz, $CDCl_3$): δ 7.67 (dd, *J* = 8.1, 6.7 Hz, 1H), 7.59-7.55 (m, 2H), 7.52-7.44 (m, 4H), 7.42 (dd, *J* = 10.2, 1.7 Hz, 1H); ^{13}C NMR (101 MHz, $CDCl_3$): δ 163.4 (d, *J* = 258.5 Hz), 148.6 (d, *J* = 8.1 Hz), 137.9 (d, *J* = 2.0 Hz), 133.7, 129.25, 129.31, 127.2, 123.4 (d, *J* = 3.2 Hz), 114.8 (d, *J* = 20.2 Hz), 114.1, 99.7 (d, *J* = 15.7 Hz).

3,3',4'-Trifluoro-[1,1'-biphenyl]-4-carbonitrile (**3f**). Yield 98% (white powder); mp 154-156°C; ^{1}H NMR (400 MHz, $CDCl_3$): δ 7.71 (dd, *J* = 8.1, 6.6 Hz, 1H), 7.43 (dd, *J* = 8.1, 1.7 Hz, 1H), 7.42-7.39 (m, 1H), 7.38-7.36 (m, 1H), 7.34-7.30 (m, 2H); ^{13}C NMR (101 MHz, $CDCl_3$): δ 163.5 (d, J_{CF} = 259.4 Hz), 151.1 (dd, J_{CF} = 250.7, 11.1 Hz), 150.8 (dd, J_{CF} = 244.8, 7.7 Hz), 146.3 (d, J_{CF} = 7.6 Hz), 135.1 (ddd, J_{CF} = 6.1, 4.0, 2.1 Hz), 134.0, 123.5 (dd, J_{CF} = 6.6, 3.6 Hz), 123.3 (d, J_{CF} = 3.3 Hz), 118.3 (d, J_{CF} = 17.4 Hz), 116.4 (d, J_{CF} = 18.3 Hz), 114.8 (d, J_{CF} = 20.6 Hz), 113.8, 100.6 (d, J_{CF} = 15.6 Hz).

4'-Chloro-3-fluoro-[1,1'-biphenyl]-4-carbonitrile (**3g**).[5] Yield 83% (white powder), mp 175-177°C; ^{1}H NMR (500 MHz, $CDCl_3$): δ 7.69 (dd, *J* = 8.1, 6.6 Hz, 1H), 7.53-7.49 (m, 2H), 7.49-7.44 (m, 3H), 7.40 (dd, *J* = 10.1, 1.7 Hz, 1H; ^{13}C NMR: δ 163.5 (d, J_{CF} = 259.2 Hz), 147.2 (d, J_{CF} = 8.2 Hz), 136.4 (d, J_{CF} = 1.8 Hz), 135.7, 133.9, 129.5, 128.4, 123.2 (d, J_{CF} = 3.5 Hz), 114.7 (d, J_{CF} = 20.4 Hz), 113.9, 100.2 (d, J_{CF} = 15.8 Hz).

3-Fluoro-3',4'-dimethoxy-[1,1'-biphenyl]-4-carbonitrile (**3h**). Yield 53% (yellow powder); mp 127-128°C; ^{1}H NMR (300 MHz, $CDCl_3$): δ 7.64 (t, *J* = 7.1 Hz, 1H), 7.45 (d, *J* = 8.0 Hz, 1H), 7.39 (d, *J* = 10.4 Hz, 1H), 7.17 (d, *J* = 7.8 Hz, 1H), 7.08 (s, 1H), 6.97 (d, *J* = 8.2 Hz, 1H), 3.94 (s, 3H), 3.96 (s, 3H); ^{13}C NMR (75 MHz, $CDCl_3$): δ 163.5 (d, J_{CF} = 258.0 Hz), 150.3, 149.5, 148.3 (d, J_{CF} = 8.2 Hz), 133.6, 130.7 (d, J_{CF} = 2.1 Hz), 122.9 (d, J_{CF} = 3.1 Hz), 119.9, 114.22, 114.20 (d, J_{CF} = 20.2 Hz), 111.6, 110.1, 99.0 (d, J_{CF} = 15.7 Hz), 56.1, 56.0.

[1,1'-Biphenyl]-3-carbonitrile (**5a**) [6]. Yield 59% (colourless oil); ^{1}H NMR (400 MHz, $CDCl_3$): δ 7.85 (t, *J* = 1.7 Hz, 1H), 7.80 (dt, *J* = 7.8, 1.5 Hz, 1H), 7.61 (dt, *J* = 7.7, 1.4 Hz, 1H), 7.57-7.55 (m, 1H), 7.54-7.53 (m, 2H), 7.50-7.44 (m, 2H), 7.43-7.40 (m, 1H); ^{13}C NMR (101 MHz, $CDCl_3$): δ 142.4, 138.8, 131.5, 130.7, 129.6, 129.1, 128.4, 127.1, 118.8, 112.9.

3',4'-Difluoro-[1,1'-biphenyl]-3-carbonitrile (**5b**) [7]. Yield 95% (white powder); mp 114-117°C; ^{1}H NMR (400 MHz, $CDCl_3$): δ 7.80 (t, *J* = 1.8 Hz, 1H), 7.75 (dt, *J* = 7.9, 1.6 Hz, 1H), 7.66 (dt, *J* = 7.7, 1.4 Hz, 1H), 7.56 (t, *J* = 7.8 Hz, 1H), 7.41-7.34 (m, 1H), 7.24-7.31 (m, 2H); ^{13}C NMR (101 MHz, $CDCl_3$): δ 150.7 (d, J_{CF} = 251.5, 12.8 Hz), 150.6 (d, J_{CF} = 250.5, 12.8 Hz), 140.4 (d, J_{CF} = 1.7 Hz), 135.9 (dd, J_{CF} = 5.9, 3.9 Hz), 131.3, 131.3, 130.5, 129.9, 123.2 (dd, J_{CF} = 6.4, 3.6 Hz), 118.5, 118.1 (d, J_{CF} = 17.5 Hz), 116.2 (d, J_{CF} = 18.1 Hz), 113.3.

4'-Chloro-[1,1'-biphenyl]-3-carbonitrile (**5c**) [8]. Yield 93% (white powder); mp 96-99°C; ^{1}H NMR (400 MHz, $CDCl_3$): δ 7.80 (t, *J* = 1.7 Hz, 1H), 7.76 (dt, *J* = 7.9, 1.6 Hz, 1H), 7.63 (dt, *J* = 7.8, 1.4 Hz, 1H),

7.54 (t, *J* = 7.8 Hz, 1H), 7.50-7.42 (m, 4H); ^{13}C NMR (101 MHz, $CDCl_3$): δ 141.2, 137.3, 134.7, 131.3, 131.0, 130.5, 129.7, 129.3, 128.3, 118.6, 113.1.

3',4'-Dimethoxy-[1,1'-biphenyl]-3-carbonitrile (**5d**). Yield 98% (white powder); mp 120-123°C; ^{1}H NMR (300 MHz, $CDCl_3$): δ 7.81 (t, 1H), 7.77 (dt, *J* = 7.7, 1.4 Hz, 1H), 7.57 (dt, *J* = 6.4, 1.3 Hz, 1H), 7.50 (t, *J* = 7.6 Hz, 1H), 7.11 (dd, *J* = 8.3, 2.1 Hz, 1H), 7.06 (d, *J* = 2.0 Hz, 1H), 6.96 (d, *J* = 8.3 Hz, 1H), 3.95 (s, 3H), 3.93 (s, 3H); ^{13}C NMR (75 MHz, $CDCl_3$): δ 149.5, 149.4, 142.2, 131.6, 131.1, 130.3, 130.1, 129.5, 119.5, 118.9, 112.8, 111.6, 110.1, 56.02, 55.99.

6-(4-Chlorophenyl)picolinonitrile (**5e**). Yield 98% (white powder); mp 97-99°C; ^{1}H NMR (400 MHz, $CDCl_3$): δ 8.01-7.96 (m, 2H), 7.93-7.89 (m, 2H), 7.63 (dd, *J* = 6.9, 1.7 Hz, 1H), 7.51-7.44 (m, 2H); ^{13}C NMR (101 MHz, $CDCl_3$): δ 157.7, 137.9, 136.5, 135.6, 133.9, 129.2, 128.3, 126.8, 123.2, 117.3.

6-(3,4-Dimethoxyphenyl)picolinonitrile (**5f**). Yield 92% (yellow powder); mp 125-128°C; ^{1}H NMR (400 MHz, $CDCl_3$): δ 7.90 (dd, *J* = 8.2, 1.0 Hz, 1H), 7.83 (t, *J* = 7.8 Hz, 1H), 7.70 (d, *J* = 2.1 Hz, 1H), 7.60-7.50 (m, 2H), 6.96 (d, *J* = 8.4 Hz, 1H), 4.01 (s, 3H), 3.95 (s, 3H); ^{13}C NMR (101 MHz, $CDCl_3$): δ 158.5, 151.0, 149.5, 137.5, 133.6, 130.0, 126.0, 122.8, 119.7, 117.6, 111.1, 109.9, 56.1, 56.0.

3',4,4'-Trifluoro-[1,1'-biphenyl]-2-carbonitrile (**7a**). Yield 70% (white powder); mp 119-121°C; ^{1}H NMR (400 MHz, $CDCl_3$): δ 7.49-7.44 (m, 2H), 7.41-7.30 (m, 2H), 7.29-7.25 (m, 2H); ^{13}C NMR (101 MHz, $CDCl_3$): δ 161.6 (d, J_{CF} = 251.5 Hz), 150.6 (dd, J_{CF} =251.5 , 55.4 Hz), 150.5 (dd, J_{CF} = 251.5 , 55.5 Hz), 139.7 (dd, J_{CF} = 3.9, 1.7 Hz), 134.0 (dd, J_{CF} = 6.2, 4.0 Hz), 132.0 (d, J_{CF} = 8.2 Hz), 125.2 (dd, J_{CF} = 6.6, 3.8 Hz), 120.8 (d, J_{CF} = 21.1 Hz), 120.4 (d, J_{CF} = 24.8 Hz), 118.1 (d, J_{CF} = 18.05), 117.9 (d, J_{CF} = 17.7 Hz), 117.0 (d, J_{CF} = 2.7 Hz), 112.7 (d, J_{CF} = 9.4 Hz).

4-Fluoro-3',4'-dimethoxy-[1,1'-biphenyl]-2-carbonitrile (**7b**). Yield 50% (yellow powder); mp 127-129°C; ^{1}H NMR (300 MHz, $CDCl_3$): δ 7.49 (dd, *J* = 8.7, 5.3 Hz, 1H), 7.43 (dd, *J* = 8.1, 2.6 Hz, 1H), 7.34 (td, *J* = 8.3, 2.7 Hz, 1H), 7.12-7.04 (m, 2H), 6.97 (d, *J* = 8.1 Hz, 1H), 3.93 (s, 3H), 3.94 (s, 3H); ^{13}C NMR (75 MHz, $CDCl_3$): δ 160.9 (d, J_{CF} = 249.6 Hz), 149.7, 149.0, 141.8 (d, J_{CF} = 3.6 Hz), 131.8 (d, J_{CF} = 8.1 Hz), 129.7, 121.4, 120.5 (d, J_{CF} = 21.2 Hz), 120.2 (d, J_{CF} = 24.6 Hz). 117.8 (d, J_{CF} = 2.8 Hz), 112.3 (d, J_{CF} = 9.2 Hz), 111.9, 111.3, 56.1, 56.0.

Spectroscopic data for compounds ***12***

2-(Hydroxy(phenyl)methyl)acrylonitrile (**12a**) [34]. Yield 96% (colourless oil): ^{1}H NMR (300 MHz, $CDCl_3$): δ 7.61-7.02 (m, 5H), 5.97 (d, *J* = 1.5 Hz, 1H), 5.89 (d, *J* = 1.3 Hz, 1H), 5.12 (s, 1H), 3.56 (s, 1H); ^{13}C NMR (75 MHz, $CDCl_3$) :δ 139.1, 130.2, 128.8, 126.5, 126.1, 117.0, 73.8.

2-((2-Bromophenyl)(hydroxy)methyl)acrylonitrile (**12b**) [9]. Yield 64% (colourless oil); ^{1}H NMR (300 MHz, $CDCl_3$): δ 7.60-7.52 (m, 2H), 7.38 (td, *J* = 7.6, 1.3 Hz, 1H), 7.20 (td, *J* = 7.7, 1.8 Hz, 1H), 6.06-6.01 (m, 2H), 5.67 (s, 1H), 3.27 (s, 1H); ^{13}C NMR (75 MHz, $CDCl_3$): δ 138.0, 133.0, 131.7, 130.3, 128.3, 128.1, 124.5, 122.7, 116.7, 72.5.

2-(Hydroxy(4-methoxyphenyl)methyl)acrylonitrile (**12c**) [10]. Yield 88% (colourless oil); ^{1}H NMR (300 MHz, $CDCl_3$): δ 7.32-7.16 (m, 2H), 6.94-6.73 (m, 2H), 6.02 (d, *J* = 1.6 Hz, 1H), 5.94 (d, *J* = 1.4 Hz, 1H), 5.14 (s, 1H), 3.76 (s, 3H), 3.33-3.28 (m, 1H); ^{13}C NMR (75 MHz, $CDCl_3$) :δ 159.8, 131.4, 129.6, 127.9, 126.4, 117.1, 114.2, 73.5, 55.3.

2-((4-Chlorophenyl)(hydroxy)methyl)acrylonitrile (**12d**).[11] Yield 95% (colourless oil); ^{1}H NMR (300 MHz, $CDCl_3$): δ 7.37-7.31 (m, 2H), 7.30-7.24 (m, 2H) 6.05 (d, *J* = 1.5 Hz, 1H), 5.98 (d, *J* = 1.1 Hz, 1H), 5.20 (s, 6H), 3.34 (s, 1H); ^{13}C NMR (70 MHz, $CDCl_3$): δ 137.7, 134.4, 130.6, 128.9, 127.9, 125.8, 116.8, 73.2.

2-((3-Bromophenyl)(hydroxy)methyl)acrylonitrile (**12e**) [50]. Yield 92% (colourless oil); ^{1}H NMR (300 MHz, $CDCl_3$): δ 7.55-7.36 (m, 2H), 7.34-7.14 (m, 2H), 6.05 (d, *J* = 1.5 Hz, 1H), 5.99 (d, *J* = 1.1 Hz, 1H), 5.17 (s, 1H), 3.58 (d, *J* = 4.7 Hz, 1H); ^{13}C NMR : δ 141.4, 131.8, 130.8, 130.4, 129.4, 125.5, 125.2, 122.8, 116.7, 73.1.

2-(Hydroxy(3,4,5-trimethoxyphenyl)methyl)acrylonitrile (**12f**).[12] Yield 60% (white powder); mp 100-105°C; ^{1}H NMR (300 MHz, $CDCl_3$): (60% yield): (white powder) δ 6.56 (s, 2H), 6.08 (d, *J* = 1.5 Hz, 1H), 5.98 (d, *J* = 1.2 Hz, 1H), 5.17 (d, *J* = 3.5 Hz, 1H), 3.82 (s, 6H), 3.79 (s, 3H), 3.77 (d, *J* = 3.6 Hz, 1H); ^{13}C NMR (75 MHz, $CDCl_3$) :δ 153.2, 137.6, 135.4, 129.8, 126.3, 117.1, 103.4, 74.0, 60.8, 56.1.

Spectroscopic data for compounds ***17***

4-(3-(Cyclohexylamino)imidazo[1,2-*a*]pyridin-2-yl)benzonitrile (**17a**). Yield 25% (yellow powder); mp 189-193°C; ^{1}H NMR (400 MHz, $CDCl_3$): δ 8.32-8.18 (m, 2H), 8.05 (dt, *J* = 6.9, 1.2 Hz, 1H), 7.75-7.65 (m, 2H), 7.53 (dt, *J* = 9.1, 1.1 Hz, 1H), 7.17 (ddd, *J* = 9.1, 6.6, 1.3 Hz, 1H), 6.81 (td, *J* = 6.8, 1.1 Hz, 1H), 3.08 (br s, 1H), 2.94 (m, 1H), 1.89-1.54 (m, 5H), 1.33-1.09 (m, 5H); ^{13}C NMR (101 MHz, $CDCl_3$): δ 141.9, 139.1, 134.5, 132.2, 127.2, 126.2, 124.8, 122.7, 119.2, 117.7, 112.2, 110.3, 57.0, 34.3, 25.6, 24.8.

4-(3-(Cyclohexylamino)-6-methylimidazo[1,2-*a*]pyridin-2-yl)benzonitrile (**17b**). Yield 21% (yellow powder); mp 230-240°C; ^{1}H NMR (400 MHz, $CDCl_3$): δ 8.40-8.15 (m, 2H), 7.92-7.78 (m, 1H), 7.77-7.63 (m, 2H), 7.50-7.43 (m, 1H), 7.14-6.96 (m, 1H), 3.03-2.97 (m, 2H), 2.48-2.33 (m, 3H), 1.91-1.56 (m, 5H), 1.39-1.13 (m, 5H); ^{13}C NMR (101 MHz, $CDCl_3$): δ 141.1, 139.3, 134.6, 132.2, 128.0, 127.1, 125.8, 121.9, 120.2, 119.3, 117.1, 110.1, 57.0, 34.3, 25.7, 24.9, 18.5.

4-(3-(Cyclohexylamino)-5-methylimidazo[1,2-*a*]pyridin-2-yl)benzonitrile (**17d**). Yield 25% (yellow oil); ^{1}H NMR (400 MHz, $CDCl_3$): δ 8.18 (d, *J* = 8.4 Hz, 2H), 7.69 (d, *J* = 8.4 Hz, 2H), 7.41 (d, *J* = 9.0 Hz, 1H), 7.04 (dd, *J* = 9.0, 6.8 Hz, 1H), 6.46 (d, *J* = 6.8 Hz, 1H), 3.07 (s, 1H), 2.92 (s, 1H), 2.82-2.72 (m, 1H), 1.76-1.47 (m, 5H) , 1.10-1.06 (m, 5H); ^{13}C NMR (101 MHz, $CDCl_3$): δ 143.7, 139.8, 137.1, 136.2, 132.1, 128.0, 127.5, 124.9, 119.3, 116.2, 114.0, 110.4, 59.2, 33.3, 25.7, 24.9, 20.0.

4-(5-Bromo-3-(cyclohexylamino)imidazo[1,2-*a*]pyridin-2-yl)benzonitrile (**17e**). Yield 42% (yellow powder); mp 165-168°C ; ^{1}H NMR (400 MHz, $CDCl_3$): δ 8.44 (d, *J* = 8.5 Hz, 2H), 7.69 (d, *J* = 8.5 Hz, 2H), 7.53 (dd, *J* = 8.4 Hz , 1.7 Hz, 1H), 7.07-6.90 (m, 2H), 3.72 (d, *J* = 4.0 Hz, 1H), 2.96-2.86 (m, 1H), 1.73-1.50 (m, 5H), 1.26-1.05 (m, 5H); ^{13}C NMR (101 MHz, $CDCl_3$): δ 144.4, 139.1, 137.2, 132.0, 128.4, 128.1, 124.9, 119.3, 118.9, 117.7, 111.8, 110.6, 59.4, 33.0, 25.8, 24.9.

4-(3-(Butylamino)imidazo[1,2-*a*]pyridin-2-yl)benzonitrile (**17f**). Yield 17% (yellow powder); mp 124-126°C; ^{1}H NMR (400 MHz, $CDCl_3$): δ 8.18 (d, *J* = 8.2 Hz, 2H), 8.02 (d, *J* = 6.9 Hz, 1H), 7.69 (d, *J* = 8.2 Hz, 2H), 7.54 (d, *J* = 9.1 Hz, 1H), 7.17 (ddd, *J* = 9.1, 6.6, 1.3 Hz, 1H), 6.83 (t, *J* = 6.7 Hz 1H), 3.11-3.02 (m, 3H), 1.70-1.50 (m, 2H), 1.48-1.38 (m, 2H), 0.93 (t, *J* = 7.3 Hz, 3H); ^{13}C NMR (101 MHz, $CDCl_3$): δ 141.9, 139.1, 133.8, 132.3, 127.5, 127.1, 124.7, 122.4, 119.2, 117.9, 112.2, 110.3, 48.1, 32.9, 20.2, 13.9.

4-(3-(Isopropylamino)imidazo[1,2-a]pyridin-2-yl)benzonitrile (**17g**). Yield 22% (yellow powder); mp 122-124°C; ^{1}H NMR (300 MHz, $CDCl_3$): δ 8.32-8.18 (m, 2H), 8.07 (dt, *J* = 6.9, 1.2 Hz, 1H), 7.77-7.63 (m, 2H), 7.54 (dt, *J* = 9.2, 1.1 Hz, 1H), 7.18 (ddd, *J* = 9.1, 6.6, 1.3 Hz, 1H), 6.82 (td, *J* = 6.8, 1.2 Hz, 1H), 3.51-3.32 (m, 1H), 3.07 (d, *J* = 4.5 Hz, 1H), 1.12 (d, *J*= 6.2 Hz, 6H); ^{13}C NMR (75 MHz, $CDCl_3$): δ 142.0, 139.1, 134.9, 132.2, 127.2, 126.3, 124.8, 122.7, 119.2, 117.7, 112.2, 110.2, 49.2, 23.4.

4. -(3-(*tert*-Butylamino)imidazo[1,2-*a*]pyridin-2-yl)benzonitrile (**17h**). Yield 17% (yellow powder); mp 170-173°C ; ^{1}H NMR (400 MHz, $CDCl_3$): δ 8.28-8.03 (m, 3H), 7.82-7.63 (m, 2H), 7.54 (d, *J* = 9.0, 1H), 7.18 (ddd, *J* = 9.2, 6.5, 1.3 Hz, 1H), 6.81 (td, *J* = 6.8, 1.2 Hz, 1H), 3.02 (s, 1H), 1.07 (s, 9H); ^{13}C NMR (101 MHz, $CDCl_3$): δ 142.5, 140.0, 137.5, 132.0, 128.4, 124.8, 124.5, 123.4, 119.2, 117.7, 111.9, 110.5, 56.7, 30.5.

4-(3-(Pentylamino)imidazo[1,2-*a*]pyridin-2-yl)benzonitrile (**17i**). Yield 35% (yellow powder); mp 120-122°C ; ^{1}H NMR (300 MHz, $CDCl_3$): δ 8.29-8.13 (m, 2H), 8.02 (dt, *J* = 6.9, 1.2 Hz, 1H), 7.73-7.65 (m, 2H), 7.54 (dt, *J* = 9.1, 1.1 Hz, 1H), 7.18 (ddd, *J* = 9.1, 6.7, 1.3 Hz, 1H), 6.83 (td, *J* = 6.8, 1.1 Hz, 1H), 3.11 (m, 1H), 3.09-2.97 (m, 2H), 1.67-1.55 (m,2H), 1.46-1.26 (m, 4H), 1.03-0.80 (m, 3H);^{13}C NMR (75 MHz, $CDCl_3$): δ 141.9, 139.1, 133.8, 132.3, 127.5, 127.1, 124.7, 122.4, 119.2, 117.8, 112.2, 110.3, 48.3, 30.5, 29.2, 22.5, 14.0.

3-(Cyclohexylamino)-2-phenylimidazo[1,2-*a*]pyridine-5-carbonitrile (**17j**); Yield 15% (yellow powder), mp 143-145°C; ^{1}H NMR (400 MHz, $CDCl_3$): δ 8.07 (d, *J* = 7.5 Hz, 2H), 7.81 (d, *J* = 8.6 Hz, 1H), 7.47 (t, *J* = 7.6 Hz, 2H, 7.35-7.38 (m, 2H), 7.14 (t, *J* = 7.9 Hz, 1H), 3.37 (d, *J* = 4.9 Hz, 1H), 3.01 (s, 1H), 1.87-1.48 (m, 5H), 1.30-1.09 (m, 5H); ^{13}C NMR (101 MHz, $CDCl_3$): δ 141.0, 139.7, 133.4, 128.6, 128.2, 127.6, 126.7, 124.1, 122.8, 121.9, 114.2, 107.7, 57.4, 33.0, 25.7, 24.9.

2-(*sec*-Butyl)-3-(cyclohexylamino)imidazo[1,2-*a*]pyridine-5-carbonitrile (**17k**). Yield 23% (yellow oil); ^{1}H NMR (300 MHz, $CDCl_3$): δ 7.74 (dd, *J* = 8.9, 1.2 Hz, 1H), 7.32 (dd, *J* = 7.0, 1.2 Hz, 1H), 7.07 (dd, *J* = 8.9, 7.1 Hz, 1H), 3.15-2.98 (m, 1H), 2.93-2.88 (m, 1H), 2.00-1.60 (m, 9H), 1.41-1.16 (m, 7H), 0.85 (t, *J* = 7.4 Hz, 3H); ^{13}C NMR (75 MHz, $CDCl_3$): δ 147.7, 141.3, 126.0, 123.5, 122.3, 121.0, 114.3, 107.4, 58.1, 33.2, 33.2, 33.0, 29.8, 25.8, 25.1, 25.0, 20.6, 12.5.

*Spectroscopic data for compounds **18a, 18b** and **19***

2,6-*Bis*-(4-chlorophenoxy)isonicotinonitrile (**18a**). Yield 99% (white oil); ^{1}H NMR (300 MHz, $CDCl_3$) δ 7.94 (d, *J* = 8.3 Hz, 1H), 7.25–7.22 (m, 2H), 6.98–6.93 (m, 2H), 6.91–6.87 (m, 2H), 6.63 (d, *J* = 8.3 Hz, 1H); ^{13}C NMR (75 MHz, $CDCl_3$) δ 162.9, 151.1, 129.6, 122.8, 122.6, 119.9, 115.0, 112.1, 106.6.

2,6-*Bis*(4-chlorophenoxy)nicotinonitrile (**18b**). Yield 78% (white oil); ^{1}H NMR (300 MHz, $CDCl_3$) δ 7.94 (d, *J* = 8.3 Hz, 1H), 7.25–7.22 (m, 4H), 6.98–6.93 (m, 2H), 6.91–6.87 (m, 2H), 6.63 (d, *J* = 8.3 Hz, 1H); ^{13}C NMR (75 MHz, $CDCl_3$) δ 164.1, 161.1, 150.8, 145.9, 132.1, 131.1, 129.4, 123.0, 114.8, 104.8.

6-(4-Chlorophenoxy)-2-((2-cyano-5-methylphenyl)amino)nicotinonitrile (**19**). Reaction of 6-(4-chlorophenoxy)nicotinonitrile (133 mg, 0.5 mmol) with 2-amino-4-methylbenzonitrile (80 mg, 0.6 mmol) using a similar method to that described for **18a** and **18b** gave product **19** (133 mg, 87%) as a yellow oil. ^{1}H NMR (300MHz, $CDCl_3$): δ 7.94 (d, *J* = 8.3 Hz, 1H), 6.63 (d, *J* = 8.3 Hz, 1H), 7.26–7.21 (m, 2H), 6.98–6.94 (m, 1H), 6.91–6.86 (m, 1H); ^{13}C NMR (75 MHz, $CDCl_3$) δ 160.1, 154.9, 151.2, 145.2, 143.9, 141.7, 132.2, 131.2, 129.8, 127.9, 123.8, 123.6, 120.1, 117.2, 115.4, 106.0, 98.9, 22.2.

*Spectroscopic data for amide products **8**, **9**, **13**, **22**, **23** and **25***

[1,1'-Biphenyl]-4-carboxamide (**8a**): [13] Yield 22% (white powder); ^{1}H NMR (400 MHz, DMSO): δ 8.02 (br s, 1H), 7.97 (d, *J* = 8.4 Hz, 2H), 7.77-7.70 (m, 4H), 7.49 (t, *J* = 7.5 Hz, 2H), 7.40 (t, *J* = 7.3 Hz, 1H, 7.38 (br s, 1H); ^{13}C NMR (101 MHz, $CDCl_3$): δ 167.5, 142.7, 139.2, 133.1, 129.0, 128.2, 128.0, 126.9, 126.4.

3',4'-Difluoro-[1,1'-biphenyl]-4-carboxamide (**8b**). Yield 31% (white powder); ^{1}H NMR (500 MHz, DMSO-d_6): δ 8.04 (br s, 1H), 7.97 (d, *J* = 8.5 Hz, 2H), 7.86 (ddd, *J* = 12.2, 7.7, 2.2 Hz, 1H), 7.78 (d, *J* = 8.5 Hz, 2H), 7.61 (dddd, *J* = 7.9, 4.5, 2.3, 1.1 Hz, 1H), 7.57-7.50 (m, 1H), 7.41 (br s, 1H); ^{13}C NMR (125 MHz, DMSO-d_6): δ 167.3, 150.6 (dd, J_{CF} = 48.3, 12.7 Hz), 148.6 (dd, J_{CF} = 49.8, 12.7 Hz), 140.4, 136.8 (dd, J_{CF} = 6.2, 3.7 Hz), 133.5, 128.2, 126.6, 123.7 (dd, J_{CF} = 6.5, 3.2 Hz), 117.9 (d, J_{CF} = 17.2 Hz), 115.9 (d, J_{CF} = 17.9 Hz).

4'-Chloro-[1,1'-biphenyl]-4-carboxamide (**8c**).[14] Yield 42% (white powder); ^{1}H NMR (500 MHz, DMSO-d_6): δ 8.03 (br s, 1H), 7.99-7.93 (m, 2H), 7.79-7.71 (m, 4H), 7.57-7.51 (m, 2H), 7.40 (br s, 1H); ^{13}C NMR (125 MHz, $CDCl_3$) :δ 167.4, 141.4, 138.0, 133.4, 132.9, 129.0, 128.7, 128.2, 126.4.

3-Fluoro-[1,1'-biphenyl]-4-carboxamide (**8e**). Yield 13% (yellow powder); ^{1}H NMR (400 MHz, DMSO-d_6): δ 7.76-7.73 (m, 3H), 7.70 (br s, 1H), 7.65 (br s, 1H), 7.63-7.57 (m, 2H), 7.52-7.48 (m, 2H), 7.45-7.40 (m, 1H); ^{13}C NMR (101 MHz, DMSO-d_6): δ 164.9 (d, J_{CF} = 1.4 Hz), 159.8 (d, J_{CF} = 249.2 Hz), 144.4 (d, J_{CF} = 8.4 Hz), 137.9 (d, J_{CF} = 1.8 Hz), 130.9 (d, J_{CF} = 3.5 Hz), 129.1, 128.6, 126.9, 122.4 (d, J_{CF} = 3.0 Hz), 122.3 (d, J_{CF} = 14.4 Hz), 114.0 (d, J_{CF} = 23.9 Hz).

3,3',4'-Trifluoro-[1,1'-biphenyl]-4-carboxamide (**8f**). Yield 23% (white powder); ^{1}H NMR (500 MHz, DMSO-d_6): (23% yield): (White powder): δ 7.91 (ddd, *J* = 12.2, 7.7, 2.3 Hz, 1H), 7.74 (t, *J* = 7.9 Hz, 2H), 7.68 (dd, *J* = 12.1, 1.7 Hz, 1H), 7.64-7.62 (m, 2H), 7.58-7.52 (m, 1H); ^{13}C NMR (125 MHz, DMSO-d_6): δ 164.8 (d, J_{CF} = 1.26 Hz) , 159.7 (d, J_{CF} = 249.4 Hz), 149.8 (dd, J_{CF} = 245.7, 12.5 Hz), 146.7 (dd, J_{CF} = 248.2, 12.8 Hz), 142.0 (d, J_{CF} = 8.5 Hz), 135.4, 130.9 (d, J_{CF} = 3.5 Hz), 123.9 (dd, J_{CF} = 6.7, 3.3 Hz), 122.8 (d, J_{CF} = 14.4 Hz), 122.5 (d, J_{CF} = 3.0 Hz), 118.1 (d, J_{CF} = 17.2 Hz), 116.2 (d, J_{CF} = 18.1 Hz), 114.3 (d, J_{CF} = 24.5 Hz).

4'-Chloro-3-fluoro-[1,1'-biphenyl]-4-carboxamide (**8g**). Yield 15% (yellow powder); ^{1}H NMR (400 MHz, DMSO-d_6): δ 7.81-7.77 (m, 2H), 7.75 (t, *J* = 8.0 Hz, 1H), 7.72 (br s, 1H), 7.68-7.59 (m, 2H), 7.57-7.53 (m, 2H); ^{13}C NMR (101 MHz, DMSO-d_6): δ 165.2 (d, J_{CF} = 1.4 Hz), 160.0 (d, J_{CF} = 249.5 Hz), 143.3 (d, J_{CF} = 8.5 Hz), 136.9, 133.7, 131.2 (d, J_{CF} = 3.4 Hz), 129.3, 128.9, 122.7 (d, J_{CF} = 3.0 Hz), 122.7 (d, J_{CF} = 14.3 Hz), 114.3 (d, J_{CF} = 24.2 Hz).

[1,1'-Biphenyl]-3-carboxamide (**9a**). Yield 20% (white powder); ^{1}H NMR (400 MHz, DMSO-d_6): δ 8.16 (t, *J* = 1.7 Hz, 1H), 8.09 (d, *J* = 9.2 Hz, 1H), 7.90-7.84 (m, 1H), 7.82 (dd, *J* = 7.9, 1.6 Hz, 1H), 7.77-7.69 (m, 2H), 7.58-7.47 (m, 3H), 7.41 (dt, *J* = 8.1, 5.2 Hz, 2H); ^{13}C NMR (101 MHz, DMSO-d_6): δ 167.8, 140.1, 139.6, 134.9, 129.4, 129.0, 128.9, 127.7, 126.8, 126.6, 125.7.

3',4'-Difluoro-[1,1'-biphenyl]-3-carboxamide (**9b**). Yield 42% (white powder); ^{1}H NMR (500 MHz, DMSO-d_6): δ 8.16 (t, *J* = 1.7 Hz, 1H), 8.10 (br s, 1H), 7.90-7.83 (m, 3H), 7.61-7.60 (m, 1H), 7.59-7.52 (m, 2H), 7.46 (br s, 1H); ^{13}C NMR (126 MHz, $CDCl_3$): δ 167.6, 149.6 (dd, J_{CF} = 247.0 , 67.9 Hz), 149.5 (dd, J_{CF} = 247.0, 69.2 Hz), 137.9, 137.2 (dd, J_{CF} = 6.3, 3.6 Hz, C8), 135.0, 129.4, 129.1, 127.2, 125.6, 123.6 (dd, J_{CF} = 6.5, 3.3 Hz), 118.0 (d, J_{CF} = 17.2 Hz), 115.9 (d, J_{CF} = 17.8 Hz).

4'-Chloro-[1,1'-biphenyl]-3-carboxamide (**9c**). Yield 11% (White powder); ^{1}H NMR (500 MHz, DMSO-*d*$_6$): δ 8.16 (t, *J* = 1.9 Hz, 1H), 8.11 (br s, 1H), 8.08 (br s, 1H), 7.79-7.76 (m, 2H), 7.58-7.53 (m, 3H), 7.47-7.39 (m, 2H); ^{13}C NMR (126 MHz, DMSO-*d*$_6$) δ 167.6 , 138.7 , 138.4 , 135.0, 133.9 , 132.6 , 130.5 , 128.9 , 128.6 , 126.5 , 125.6.

6-(4-Chlorophenyl)picolinamide (**9e**). Yield 5% (white powder); ^{1}H NMR (400 MHz, DMSO-*d*$_6$): δ 8.39-8.31 (m, 3H), 8.18 (dd, *J* = 7.9, 1.1 Hz, 1H), 8.06 (t, *J* = 7.8 Hz, 1H), 7.99 (dd, *J* = 7.6, 1.0 Hz, 1H), 7.70 (br s, 1H), 7.58-7.51 (m, 2H); ^{13}C NMR: δ 166.2, 153.8, 150.3, 139.0, 136.5, 134.5, 128.94, 128.88, 122.7, 120.9.

2-(Hydroxy(phenyl)methyl)acrylamide (**13a**) [10]. Yield 84% (colourless oil); ^{1}H NMR (300 MHz, DMSO-*d*$_6$): δ 7.46 (s, 1H), 7.38-7.27 (m, 4H), 7.27-7.20 (m, 1H), 7.00 (s, 1H), 5.81 (t, *J* = 1.2 Hz, 1H), 5.70 (d, *J* = 4.9 Hz, 1H), 5.62 (t, *J* = 1.4 Hz, 1H), 5.51 (dt, *J* = 4.9, 1.2 Hz, 1H); ^{13}C NMR (75 MHz, DMSO-*d*$_6$) δ 139.2, 130.0, 128.9, 128.8, 126.5, 126.2, 117.0, 74.0.

2-((2-Bromophenyl)(hydroxy)methyl)acrylamide (**13b**). Yield 93% (white powder); ^{1}H NMR (300 MHz, DMSO-*d*$_6$): δ 7.60-7.50 (m, 2H), 7.46-7.32 (m, 2H), 7.20 (ddd, *J* = 7.9, 7.1, 2.0 Hz, 1H), 7.02 (br s, 1H), 5.84-5.83 (m , 1H), 5.80-5.73 (m, 1H), 5.70 (d, *J* = 5.8 Hz, 1H), 5.28-5.21 (m, 1H); ^{13}C NMR (75 MHz, DMSO-*d*$_6$): δ 168.7, 146.4, 141.9, 132.4, 129.1, 128.7, 127.5, 123.2, 118.6, 70.0.

2-(Hydroxy(4-methoxyphenyl)methyl)acrylamide (**13c**) [10]. Yield 57% (white powder); ^{1}H NMR (300 MHz, DMSO-*d*$_6$): δ 7.40 (br s, 1H), 7.27-7.15 (m, 2H), 6.95 (br s, 1H), 6.89-6.79 (m, 2H), 5.76 (t, *J* = 1.3 Hz, 1H), 5.62-5.51 (m, 2H), 5.43 (d, *J* = 4.7 Hz, 1H), 3.72 (s, 3H); ^{13}C NMR (75 MHz, DMSO-*d*$_6$) :δ 168.7, 158.3, 147.7, 135.2, 127.9, 116.9, 113.3, 70.7, 55.0.

2-((4-Chlorophenyl)(hydroxy)methyl)acrylamide (**13d**) [15]. Yield 61% (white powder); mp 132-134; ^{1}H NMR (500 MHz, DMSO-*d*$_6$): δ 7.45 (s, 1H), 7.38 – 7.33 (m, 2H), 7.33 – 7.26 (m, 2H), 6.99 (s, 1H), 5.81 (t, *J* = 1.1 Hz, 1H), 5.77 (d, *J* = 4.9 Hz, 1H), 5.62 (t, *J* = 1.4 Hz, 1H), 5.48 (d, *J* = 4.8 Hz, 1H); ^{13}C NMR (126 MHz, DMSO-*d*$_6$): δ 168.5, 147.1, 142.4, 131.4, 128.6, 127.9, 117.6, 70.4.

2-(Hydroxy(4-methoxyphenyl)methyl)acrylamide (**13e**) [10]. Yield 57% (white powder); ^{1}H NMR (300 MHz, DMSO-*d*$_6$): δ 7.40 (br s, 1H), 7.27-7.15 (m, 2H), 6.95 (br s, 1H), 6.89-6.79 (m, 2H), 5.76 (t, *J* = 1.3 Hz, 1H), 5.62-5.51 (m, 2H), 5.43 (d, *J* = 4.7 Hz, 1H), 3.72 (s, 3H); ^{13}C NMR (75 MHz, DMSO-*d*$_6$) :δ 168.7, 158.3, 147.7, 135.2, 127.9, 116.9, 113.3, 70.7, 55.0.

2,6-*Bis*(4-chlorophenoxy)isonicotinamide (**22a**). Yield 73% (yellow oil); ^{1}H NMR (500 MHz, DMSO-*d*$_6$) δ 9.63 (br s, 2H), 7.88 (s, 2H), 7.17 (d, *J* = 8.4 Hz, 4H), 6.75 (d, *J* = 8.3 Hz, 4H); ^{13}C NMR (126 MHz, DMSO-*d*$_6$) δ 162.8, 156.8, 150.7, 143.8, 129.6, 123.2, 122.8, 117.4.

2,6-*Bis*(4-chlorophenoxy)nicotinamide (**22b**). Yield 50%; ^{1}H NMR (500 MHz, $CDCl_3$): *δ* 8.35 (s, 1H), 8.33 (s, 1 H), 7.94-7.92 (m, 3H), 7.41-7.39 (m, 2H), 7.38-7.10 (m, 2 H), 6.92-6.90 (m, 1H), 6.77 (s, 1H), 5.96 (s, 1H); ^{13}C NMR (126 MHz, $CDCl_3$): 151.7, 151.1, 150.6, 131.3, 130.9, 130.8, 130.0, 129.9, 129.6, 122.8, 122.6, 118.9, 113.9, 110.1.

6-(4-Chlorophenoxy)-2-((2-cyano-5-methylphenyl)amino)nicotinamide (**23**). Yield 64% (light yellow solid); mp 204–205°C; ^{1}H NMR (500 MHz, DMSO-*d*$_6$) δ 9.40 (br s, 1H), 8.18 (d, *J* = 8.4 Hz), 7.51–7.45 (m, 3H), 7.43–7.40 (m, 2H), 7.31 (s, 1H), 7.26–7.21 (m, 2H), 6.91 (d, *J* = 7.9 Hz), 6.77 (d, *J* = 8.4 Hz, 1H), 2.12 (s, 3H); ^{13}C NMR (126 MHz, DMSO-*d*$_6$) δ 165.2, 159.5, 155.6, 152.2, 144.4, 143.1, 142.1, 142.3, 133.2, 129.7, 129.5, 124.7, 124.5, 123.4, 117.9, 107.9, 105.8, 101.8, 21.8.

2-(2-Cyanophenyl)acetamide (**25p**). Yield 10%; ^{1}H NMR (500 MHz; $CDCl_3$): δ 7.68-7.61(m, 1H), 7.60-7.58 (m, 1H), 7.54-7.53 (m, 1H), 7.42-7.39 (m, 1 H), 5.63 ppm (s, 1H), 5.47 ppm (s, 1H), 3.79 ppm (s, 2H); ^{13}C NMR (126 MHz, $CDCl_3$): δ 170.6, 138.5, 133.3, 132.8, 130.9, 127.9, 117.9, 112.9, 41.3.

4-Cyanatophenyl carbamate (**33**). Yield 21%; ^{1}H NMR (500 MHz, $CDCl_3$): δ 7.93-7.90 (m, 2 H), 7.78-7.75 (m, 2H), 6.00 (s, 1H), 5.69 (s, 1H); ^{13}C NMR (101 MHz, $CDCl_3$): 167.2, 137.2, 132.5, 128.0, 117.8, 115.8.

1*H*-pyrrolo[2,3-*b*]pyridine-4-carboxamide (**35**). Yield 22% (Brown powder); ^{1}H NMR (300 MHz, DMSO-*d*$_6$): δ 11.83 (s, 1H), 8.28 (d, *J* = 4.9 Hz, 1H), 8.00 (s, 1H), 7.63-7.46 (m, 3H), 7.39 (d, *J* = 4.9 Hz, 1H), 6.81 (d, *J* = 3.4 Hz, 1H); ^{13}C NMR (75 MHz, DMSO-*d*$_6$): δ 168.3, 149.7, 142.2, 133.4, 127.5, 117.4, 113.5, 100.6 .

Reference

1. Man Wang, X.Y.; Hongyu, L.; Limin, R.; Zhizhong, S.; Yanjun, H.; Wenyi C.. Nickel-catalysed Suzuki-Miyaura cross coupling of aryl halides with arylboronic acids in ionic liquids. Cat. Commun., **2015**, 58, 154-157.
2. Moradi, P.; Hajjami, M.; Valizadeh-Kakhki, F., Biochar as heterogenous support for immobilization of Pd as efficient and reusable biocatalyst in C-C coupling. *Appl. Organometallic Chem*., **2019**: *33*, e5205.
3. Reeves, E.K.; Humke, J.N.; Neufeldt, S.R. N-Heterocyclic Carbene Ligand-Controlled Chemodivergent Suzuki-Miyaura Cross Coupling., J. Org. Chem., **2019**, 84, 11799-11812.
4. Scheuermann, G. M.; Rumi, L.; Steurer, P.; Bannwarth, W.; Mulhaupt, R., Palladium nanoparticles on graphite oxide and its finctionalized graphene derivatives as highly active catalysis for the Suzuki-Miyaura coupling reaction. J. Am. Chem. Soc. **2009**, *131*, 8262-8270.
5. Li, X.; Hilgers. M.; Cunningham, M.; Chen, Z.; Trzoss, M.; Zhang, J.; Kohnen, L.; Lam, T.; Creighton C. G. C K.; Nelson, K.; Kwan, B.; Stidham, M.; Brown-Driver, V.; Shaw, K. J.; Finn, J., Structure-based design of new DHFR-based antibacterial agents: 7-aryl-2,4-diaminoquinazolines. *Bioorg. Med. Chem. Lett.* **2011**, *21*, 5171-5176.
6. Bhattacharyya, B.; Biswas, J. P.; Mishra, S.; Gogoi, N., Rapid Suzuki-Miyaura cross-coupling reaction catalyzed by zirconium carboxyphosphonate supported mixed valent Pd (0)/ Pd (II) catalyst. App. Organometallic Chem., **2019**, *33*, e5017.
7. Lacbay, C. M.; Menni, M.; Bernatchez, J. A.; Götte, M.; Tsantrizos, Y. S., Pharmacophore requirements for HIV-1 reverse transcriptase inhibitors that selectively "Freeze" the pre-translocated complex during the polymerization catalytic cycle. *Bioorg. Med. Chem*., **2018,** *26*, 1713-1726.
8. He, Q.; Wang, L.; Liang, Y.; Zhang, Z.; Wnuk, S.F. Transition-Metal-Free Cross-Coupling of Aryl Halides With Arylstannanes. J. Org. Chem., **2016**, 81, 9422-9427.
9. Cocco, M.; Garella, D.; Di Stilo, A.; Borretto, E.; Stevanato, L.; Giorgis, M.; Marini, E.; Fantozzi, R.; Miglio, G.; Bertinaria, M., Electrophilic warhead-based design of compounds preventing NLRP3 inflammasome-dependent pyroptosis. *J. Med. Chem*., **2014,** *57*, 10366-10382.
10. Kim, E. S.; Lee, H. S.; Kim, J. N., An efficient synthesis of Baylis–Hillman adducts of acrylamide: Pd-catalyzed hydration of Baylis–Hillman adducts of acrylonitrile. Tetrahedron Lett. **2009**, *50*, 6286-6289.
11. Reddy T. N.; Ravinder, M.; Bikshapathi R.; Sujitha P.; Kumar, C. G.; Rao, V. J., Design, synthesis, and biological evaluation of 4-H pyran derivatives as antimicrobial and anticancer agents. *Med. Chem. Res*., **2017**, *26*, 2832–2844.
12. Mendoza-Espinosa, D.; González-Olvera, R.; Osornio, C.; Negrón-Silva, G.; Santillan, R. Versatile O- and S- functionalized 1,2,3-triazoliums: ionic liquids for the Baylis-Hillman reaction and ligand precursors for stable MIC-transition metal complexes. *New J. Chem*., **2015**, *39*, e1039.
13. Uchida, K.; Togo, H., Transformation of aromatic bromides into aromatic nitriles with n-BuLi, pivalonitrile, and iodine under metal cyanide-free conditions. *Tetrahedron*, **2019**, *75*, 130550.
14. Appa, R.; Prasad, S.; Lakshmidevi, J.; Bandameeda, R.; Narasimhulu, M.; Katta, V., Palladium-catalysed room-temperature Suzuki–Miyaura coupling in water extract of pomegranate ash, a bio-derived sustainable and renewable medium. *Appl. Organomet. Chem*., **2019**, *33*, e5126.
15. Jurčík, V., Wilhelm, R., An imidazolinium salt as ionic liquid for medium and strong bases. Green Chem., 2005, 7, 844.
